# An internet-based cognitive-behavioral self-management intervention for patients with hand osteoarthritis or fibromyalgia – Two randomized controlled trials

**DOI:** 10.1016/j.invent.2026.100908

**Published:** 2026-01-19

**Authors:** Jessy A. Terpstra, Sylvia van Beugen, Rosalie van der Vaart, Roxy A. van Eersel, Elise Dusseldorp, Margreet Kloppenburg, Andrea W.M. Evers

**Affiliations:** a Health, Medical and Neuropsychology Unit, Institute of Psychology, Leiden University, PO Box 9555, 2300 RB, Leiden, the Netherlands; b Department of Rheumatology, Leiden University Medical Center, C1-R, PO Box 9600, 2300 RC, Leiden, the Netherlands; c Centre of Expertise Health Innovation, The Hague University of Applied Sciences, Johanna Westerdijkplein 75, 2521 EN, The Hague, the Netherlands; d Department of Psychiatry, Leiden University Medical Center, B1-P, PO Box 9600, 2300 RC, Leiden, the Netherlands; e Medical Delta, Huismansingel 4, 2629 JH, Delft, the Netherlands

**Keywords:** Cognitive behavioral therapy, Self-management, Chronic pain, Fibromyalgia, Hand osteoarthritis, Rheumatic diseases

## Abstract

**Background:**

Rheumatic and musculoskeletal diseases have a high burden. We aimed to evaluate the effectiveness of a therapist-guided internet-based cognitive-behavioral therapy (iCBT) on pain coping and secondary physical, psychological, and disease impact outcomes in hand osteoarthritis and fibromyalgia.

**Method:**

Two single-center, parallel-group, superiority randomized controlled trials were performed. In one study, 70 adults with hand osteoarthritis visiting a Dutch hospital were randomized to care-as-usual or care-as-usual plus iCBT (each group *n* = 35; ClinicalTrials.gov: NCT05872633). In another study, 70 adults with fibromyalgia visiting a Dutch fibromyalgia-specialized center were randomized to iCBT (*n* = 34) or a waitlist (*n* = 36; ClinicalTrials.gov: NCT06322485). Standardized self-report questionnaires were used at baseline, post-intervention, 6-week, and 3-month follow-up and analyzed with statistician-masked intention-to-treat linear mixed models. The primary endpoint was pain coping at post-intervention.

**Results:**

In patients with hand osteoarthritis (mean age = 62.4 ± 7.6), no between-group effect on pain coping was found at post-intervention (*p* = 0.187; Cohen's *d* = 0.14), while a small to medium effect favored iCBT at 6-week follow-up (*p* = 0.039; *d* = 0.41). In patients with fibromyalgia (mean age = 46.4 ± 11.8), a medium to large improvement in pain coping favoring iCBT at post-intervention (*p* = 0.003; *d* = 0.60) was not sustained at follow-up. Between-group small to large improvements were found in secondary outcomes (e.g., well-being, osteoarthritis disability, fibromyalgia pain and impact), predominantly at 3-month follow-up (*p* ≤ 0.047; 0.30 ≤ *d* ≤ 0.98).

**Conclusions:**

ICBT improved pain coping in fibromyalgia at the primary endpoint, whereas the hand osteoarthritis trial was negative at the primary endpoint. Exploratory secondary findings suggest potential benefits for both conditions but warrant replication, particularly in subgroups with a high disease impact.

## Highlights

• Internet-based CBT was studied in hand osteoarthritis and fibromyalgia.
• The primary outcome was between-group pain coping at post-treatment.
• Fibromyalgia: between-group pain coping improved post-treatment, not at follow-up.
• Hand osteoarthritis: no post-treatment pain coping effect; modest 6-week effect.
• Exploratory benefits were found in secondary outcomes, mostly at 3-month follow-up.

## 1 Introduction

Rheumatic and musculoskeletal diseases impose a high burden, marked by pain, loss of strength and mobility, and physical disability (Salaffi et al., 2019; Sebbag et al., 2019). These problems reduce health-related quality of life (HR-QoL), a multidimensional evaluation of the impact of disease and treatment on patients' functioning and well-being (Sitlinger and Zafar, 2018), and generate substantial direct and indirect costs (Hawker, 2019). While there is currently no cure for most rheumatic and musculoskeletal diseases, the symptoms can be managed (Safari et al., 2020). For osteoarthritis, analgesics and non-pharmacological modalities such as self-management, education, and provision of lifestyle advice are recommended by guidelines (Terpstra et al., 2022b). For fibromyalgia, a graduated approach with an initial focus on patient education and non-pharmacological treatment such as exercise is recommended (Macfarlane et al., 2017). Further therapies (e.g., psychological therapies, including cognitive-behavioral therapy, and pharmacotherapy) can be added based on the patient's needs (Macfarlane et al., 2017).

Cognitive-behavioral therapy (CBT) has been utilized as an effective self-management intervention in chronic pain conditions to improve patients' pain coping and HR-QoL (Cunningham and Kashikar-Zuck, 2013). Pain coping refers to the deliberate strategies individuals use to manage the stress and impact of chronic pain (Jensen et al., 1991), which can be maladaptive or adaptive (Peres and Lucchetti, 2010). Adaptive pain coping is associated with reduced pain (Rini et al., 2021) and lower pain interference and psychological distress (Scheidegger et al., 2023), whereas maladaptive coping is linked to increased pain, depression, and anxiety (Miller-Matero et al., 2016). CBT targets maladaptive beliefs and behaviors related to illness and pain using structured cognitive and/or behavioral exercises (Cunningham and Kashikar-Zuck, 2013). However, patients with chronic pain can have difficulty accessing these interventions due to treatment costs and limited treatment availability, limited mobility, and stigma associated with psychological treatment (Ehde et al., 2014). Internet-based cognitive-behavioral therapy (iCBT) has noted advantages that minimize these barriers.

Systematic reviews indicate significant improvements after iCBT for chronic pain in psychological, physical, and disease impact outcomes (Gandy et al., 2022; Terpstra et al., 2021, Terpstra et al., 2022a). For example, a meta-analysis on guided iCBT for chronic pain found that iCBT was more effective than passive control conditions for psychological (standardized mean differences [SMDs] = 0.34–0.47), physical (SMDs = 0.26–0.29), and impact on daily life outcomes (SMDs = 0.38–0.41) (Terpstra et al., 2022a). Another meta-analysis by Gandy et al. (2022) on guided and unguided iCBT for chronic pain found that iCBT was more effective than passive and active control conditions for depression, anxiety, self-efficacy, pain catastrophizing, pain intensity, and interference/disability (Hedges' *g* = 0.27–0.43). Prior research on the effectiveness of therapist guidance in iCBT for chronic pain showed mixed results (Buhrman et al., 2016; Vugts et al., 2018). However, the more recent meta-analysis by Gandy et al. (2022) found that guided iCBT had significantly greater effect sizes for anxiety, pain intensity, and interference/disability (*g =* 0.33–0.39), than unguided iCBT (*g =* 0.16–0.20). Another meta-analysis demonstrated an association between the therapeutic relationship and outcome (*r* = 0.203) in internet-based interventions (Kaiser et al., 2021).

While evidence for the effectiveness of iCBT for chronic pain is accumulating, specific populations such as patients with hand osteoarthritis and fibromyalgia remain understudied. A single-arm interventional study reported that digital rehabilitation with CBT components for hand and wrist pain, including hand osteoarthritis, improved pain, disability, analgesic intake, surgery intention, work productivity, and mental health outcomes (Costa et al., 2022). However, to date, the effects of therapist-guided iCBT for this group specifically have not been reported. Similarly, few studies exist on guided iCBT for fibromyalgia and the studies that have been performed contain some type of risk of bias (e.g., detection and/or reporting bias; Terpstra et al., 2021), underscoring the need for more rigorous research.

Compared to other chronic pain conditions frequently included in iCBT trials, such as generalized musculoskeletal or chronic back pain (Terpstra et al., 2022a), fibromyalgia and hand osteoarthritis represent unique characteristics that may influence iCBT outcomes. Fibromyalgia involves widespread pain, fatigue, cognitive symptoms, and elevated psychological distress (Macfarlane et al., 2017), leading to substantial disability and reduced quality of life (Gálvez-Sánchez et al., 2019). Hand osteoarthritis, though localized, can also impair daily functioning and is characterized by hand pain, limited hand mobility, stiffness, and weakness (Terpstra et al., 2022b). A qualitative study in hand osteoarthritis highlighted the importance of understanding patients' illness and treatment beliefs, aligning with the cognitive targets of iCBT (Hill et al., 2011). Given limited pharmacological options for both conditions (Macfarlane et al., 2017; Terpstra et al., 2022b), iCBT may offer a promising self-management approach (Terpstra et al., 2021).

This paper evaluates the effectiveness of a therapist-guided iCBT, Master Your Pain, in two studies involving outpatients with hand osteoarthritis and fibromyalgia. These two trials are presented together to evaluate the effectiveness of iCBT across two clinically distinct chronic pain conditions, one localized and one widespread, both of which are underrepresented in research and may benefit from accessible psychological treatment options. Master Your Pain was adapted from E-coach, a tailored iCBT for chronic somatic conditions showing positive effects in rheumatoid arthritis (Ferwerda et al., 2017) and psoriasis (van Beugen et al., 2016). Specifically, in rheumatoid arthritis, E-coach showed larger improvements than standard care in depressed mood, negative mood, anxiety, and role limitations due to emotional problems (*d* = 0.38–0.54) (Ferwerda et al., 2017). In psoriasis, E-coach showed larger improvements than standard care in physical functioning and impact on daily activities (*d* = 0.35–0.36), but not in psychological functioning (van Beugen et al., 2016). In contrast to the broader E-coach program, Master Your Pain has a select number of modules aimed at optimizing pain coping. For hand osteoarthritis and fibromyalgia, we hypothesized that the iCBT group would show larger improvements than control conditions (care-as-usual or a waitlist, respectively) in the primary outcome of pain coping and in secondary physical, psychological, and impact on daily life outcomes. Evaluating the effectiveness of iCBT across a broad range of outcomes in these two conditions may provide insights for both general and condition-specific clinical recommendations.

## 2 Patients and methods

### 2.1 Design

Two stratified, parallel-group, superiority randomized controlled trials (RCTs) were conducted in The Netherlands. One study took place at the rheumatology clinic of Leiden University Medical Center and one at Fibrocentrum, a fibromyalgia-specialized psychological treatment center. Patients contributed to the intervention and clinical trial design, pilot-testing, and user-friendliness optimization of a previous iCBT for chronic somatic conditions (Ferwerda et al., 2017; van Beugen et al., 2016), from which the current iCBT was adapted. The studies complied with the Declaration of Helsinki, relevant laws, and institutional guidelines. Medical Ethics Committee Leiden The Hague Delft approved both studies (hand osteoarthritis: NL55536.058.15, 20 April 2016; fibromyalgia: NL58130.058.16, 30 November 2016). All patients provided written informed consent and privacy rights were observed.

The hand osteoarthritis (NTR6266) and fibromyalgia (NTR6267) studies were initially preregistered with the Dutch National Trial Registry, which was later discontinued. Study information was automatically but incorrectly transferred to a new registry (Landelijk Trial Register, later renamed OMON), along with study information from another defunct portal (ToetsingOnline). This resulted in duplicate and erroneous entries (e.g., incorrect control group descriptions), which could not be corrected. To ensure accuracy, both studies were reregistered on ClinicalTrials.gov using the original, correct information (hand osteoarthritis: NCT05872633, 14 May 2023, https://clinicaltrials.gov/study/NCT05872633; fibromyalgia study: NCT06322485, 14 March 2024, https://clinicaltrials.gov/study/NCT06322485). Detailed analysis plans were preregistered before statistical analyses. Trial results will be posted to DataverseNL after publication.

### 2.2 Patients

Inclusion criteria are detailed in Table 1 (Altman et al., 1990). In the hand osteoarthritis study, adults with hand osteoarthritis visiting the rheumatology clinic at Leiden University Medical Center were invited to participate in the study by their rheumatologist, who provided a study information letter and informed consent form. A researcher subsequently confirmed eligibility.

**Table 1:** Inclusion and exclusion criteria in the hand osteoarthritis study and fibromyalgia study.

Hand osteoarthritis study	Fibromyalgia study
Inclusion criteria: (1) Diagnosed with hand osteoarthritis following the American College of Rheumatology (ACR) criteria (Altman et al., 1990). (2) Referred to the hand osteoarthritis care program. (3) Complaints from hand osteoarthritis including pain for a minimal duration of 3 months. (4) Minimum age of 18 years. (5) Fluent in Dutch. (6) Able to give informed consent. (7) Own a computer with internet access.	Inclusion criteria: (1) Diagnosed with fibromyalgia, as previously confirmed by their treating GP or a medical specialist. (2) Pain complaints with a minimal duration of 3 months. (3) Minimum age of 18 years. (4) Fluent in Dutch. (5) Able to give informed consent. (6) Own a computer with internet access.

Exclusion criteria: (1) Difficulties with communication (e.g., due to analphabetism) and internet literacy (e.g., not having at least “reasonable” self-assessed skills in using the internet). (2) Severe psychiatric comorbidities which interfere with the study protocol. (3) Ongoing psychological treatment elsewhere. (4) Secondary osteoarthritis due to diseases such as inflammatory rheumatic diseases, bone diseases, metabolic diseases associated with joint disease, and severe crystal arthropathies.	Exclusion criteria: (1) Difficulties with communication (e.g., due to analphabetism) and internet literacy (e.g., not having at least “reasonable” self-assessed skills in using the internet). (2) Severe physical and psychiatric comorbidities that interfere with the study protocol, such as psychosis, addiction, suicidal ideation. (3) Ongoing psychological treatment elsewhere. (4) Participation in other clinical trials. (5) Pregnancy.

In the fibromyalgia study, adults with fibromyalgia were invited to participate by their psychologist at Fibrocentrum. Study information was also displayed on the websites of Fibrocentrum and a fibromyalgia patient organization, and on Facebook, where patients could leave their contact information. A psychologist confirmed eligibility and provided an information letter and informed consent form. After providing written consent, patients in both studies received a baseline questionnaire (T1) via Qualtrics (Qualtrics, 2019). Data were collected using self-report questionnaires. Afterwards, patients were randomized to intervention or control groups.

### 2.3 Randomization and masking

Patients with hand osteoarthritis were randomized to care-as-usual plus iCBT or care-as-usual and patients with fibromyalgia were randomized to iCBT or a waitlist. A statistician generated balanced (1:1) randomization schedules in *R*, stratified by sex and age in blocks of six patients with four groups (hand osteoarthritis: male/female × <50/≥50 years; fibromyalgia: male/female × <40/≥40 years). The randomization schedules were communicated with an independent data manager, who kept them in a secured computer file. The data manager relayed each patient's assigned group to the research team via secure e-mail after a patient was enrolled by the research team. Blinding of participants and practitioners was not possible due to the study design. However, the statistician who created the randomization schedule and who was involved with statistical analyses was blinded to group allocation using masked datasets.

### 2.4 Procedures

In the hand osteoarthritis study, the intervention group first received care-as-usual, followed by iCBT. Care-as-usual in both the hand osteoarthritis intervention group and control group started with a 30-min consultation with a rheumatologist encompassing history taking and a physical examination. Subsequently, consultations with a clinical nurse and occupational therapist of 1.5 h in total took place covering the nature of osteoarthritis, chronic pain, prognosis, available treatments, joint protection, lifestyle, and assistive devices. In the fibromyalgia study, patients in the control group were waitlisted and received the intervention 6 months after baseline. Outcomes in both studies were assessed via web-based (Qualtrics) questionnaires at baseline (T1), post-intervention (T2), 6 weeks (T3), and 3 months post-intervention (T4).

#### 2.4.1 Intervention content and therapist guidance

The iCBT is detailed in Supplementary Appendix B, including Fig. S1, and comprised six text-based modules (covering an introduction, mood, activity, thoughts, social environment, relapse prevention), featuring psychoeducation, assignments, relaxation, and self-monitoring. The intervention started with one or two face-to-face intake sessions (1–1.5 h total) to set goals. Participants received weekly asynchronous therapist feedback (therapists spent ∼30 min weekly per participant) and two optional booster sessions (∼15–30 min per call) at 1 and 2.5 months post-treatment. The iCBT was delivered by trained master's-level psychologists under supervision by a senior clinical psychologist. While the protocols estimated a 12- to 16-week duration, actual length varied due to tailoring (see Section 3.1).

### 2.5 Outcomes and adverse events

The primary endpoint in both studies was the difference in change in pain coping between intervention and control groups from baseline to post-intervention. A visual analogue scale (VAS; 0–10) was utilized to assess pain coping, with higher scores indicating better pain coping. The instruction was as follows: “The next question concerns your fibromyalgia/hand osteoarthritis symptoms [adapted depending on the study]. Below is a scale on which you can indicate with a cross how well you were able to cope with the pain over the past week. On the left it says ‘very poorly’ and on the right ‘very well’. So, the better you were able to cope with the pain, the more to the right you place the mark.” VAS has shown sound psychometric properties in a rheumatoid arthritis sample undergoing routine clinical care (Sendlbeck et al., 2015; Supplementary Appendix C).

Secondary outcomes were VAS well-being and pain, hand osteoarthritis pain and disability (Australian/Canadian Hand Osteoarthritis Index [AUSCAN]; Bellamy et al., 2002), pain interference (Multidimensional Pain Inventory-Dutch Language Version [MPI-DLV]; Lousberg et al., 1999), illness cognitions (Illness Cognition Questionnaire [ICQ]; Evers et al., 2001), illness perception (Illness Perception Questionnaire-Revised [IPQ-R]; Moss-Morris et al., 2002), pain coping strategies (Pain Coping Inventory [PCI]; Kraaimaat et al., 1997), physical and mental health-related quality of life (RAND 36; Hays et al., 1993), and fibromyalgia impact (Fibromyalgia Impact Questionnaire [FIQ]; Burckhardt et al., 1991). Exploratory measures included social support, life control, and affective distress (MPI-DLV; Lousberg et al., 1999). Additional measures covered descriptive characteristics, treatment evaluation, and the therapeutic relationship (self-report questionnaires, hand osteoarthritis physical examination, Eysenck Personality Questionnaire short-form [EPQ-RSS; Sanderman et al., 2012], Internet-specific Therapeutic Relationship Questionnaire [ITRQ; Ferwerda et al., 2016]). Full descriptions are provided in Supplementary Appendix C.

Adverse events (definitions in Supplementary Appendix C) could be reported by patients, clinicians, e-coaches, and researchers and were discussed with the lead clinician (hand osteoarthritis study) and principal investigator; follow-up with patients was planned. Serious adverse events would be reported to the medical ethics committee.

### 2.6 Sample size

Previous research showed improvements in pain coping between 10 and 19% after (i)CBT for chronic pain (Boschen et al., 2016; Vallejo et al., 2015). An average 15% difference on VAS pain coping between the intervention and control group was considered relevant. For a repeated-measures analysis with four measurements and a correlation between assessment points of 0.5, 35 patients per group are required to obtain a power of 80% at α = 0.05 (calculated via G*Power; Faul et al., 2007). The sample size calculation was originally based on a repeated-measures ANOVA framework, as we had initially planned repeated-measures ANOVA before changing to more sophisticated linear mixed models (Supplementary Appendix A). Because linear mixed models generally achieve equal or greater power than repeated-measures ANOVA, the studies are unlikely to have been underpowered relative to the original calculation (Gueorguieva and Krystal, 2004; Ma et al., 2012). The assumed variance components in the power analysis (compound symmetry, correlation = 0.5) are similar compared to the variance structure estimated by the linear mixed models (which was Identity, meaning constant variance).

### 2.7 Statistical analysis

Contrary to the preregistered plan (see ClinicalTrials.gov), between-group differences in baseline characteristics were not analyzed, following CONSORT guidelines (Hopewell et al., 2025). We have retained baseline comparisons between intervention completers and non-completers to explore potential differences between these non-randomized subgroups. Change scores for primary and secondary outcomes were calculated by subtracting baseline scores from T2, T3, and T4 scores for available cases. Standardized mean differences (Cohen's *d*) between the intervention and control groups were calculated. Cohen's *d* was defined as the mean difference in change scores between groups divided by the pooled standard deviation of the change scores, with 95% confidence intervals based on the noncentral *t* distribution. Effect sizes of 0.2, 0.5, and 0.8 were considered small, medium, and large, respectively (Cohen, 1988).

To evaluate iCBT effectiveness, linear mixed models were fitted using full maximum likelihood estimation following intention-to-treat principles (ITT; i.e., including observed data from all randomized participants). Missing data from drop-outs were not imputed, as linear mixed models accommodate missingness (Gabrio et al., 2022; Peters et al., 2012). Missing data were assumed missing at random, as dropout was expected to depend on observed rather than unobserved variables (Gabrio et al., 2022). Person-mean imputation was applied at the item level when necessary to calculate scale scores (e.g., when one or two items were missing from a multi-item questionnaire at a specific time point).

The models included fixed effects of time, fixed effects of time-by-group interactions, fixed effects of age and sex covariates, and a random intercept. Time was dummy-coded with baseline as the reference (coded 0), and three dummy variables represented post-intervention (short-term), 6-week (mid-term), and 3-month follow-ups (long-term). Each dummy variable was coded 1 for the corresponding time point and 0 otherwise. The coefficients of the time dummy variables estimate mean change from baseline in the control group, and the coefficients of the time-by-group interactions estimate the additional mean change from baseline in the intervention group relative to the control group. Thus, the models test group differences in adjusted marginal means, accounting for covariates and random intercepts. The group main effect was excluded as a pre-specified, design-based choice, consistent with longitudinal data analysis literature, where baseline means can reasonably be constrained to be equal under randomization (Fitzmaurice et al., 2004, pp. 126–127). More recent work formalizes this approach under the constrained longitudinal data analysis framework, which has been shown to yield unbiased and efficient estimates of treatment effects (Coffman et al., 2016). The addition of random slopes was tested with Likelihood Ratio Tests. For each model, the variance-covariance matrix yielding the best fit was selected using restricted maximum likelihood estimation. Assumptions of normality, homoscedasticity, and linearity were checked, with transformations applied as needed. Sensitivity analyses included models without age and sex covariates and completer-only analyses with and without age and sex covariates to assess the robustness of the primary results. All additional analyses conducted are reported; all conducted analyses were prespecified.

The proportion of participants achieving a minimal clinically important improvement (MCII) was calculated, in line with previous literature (≥10% on VAS pain coping at T2; Boschen et al., 2016; Dworkin et al., 2008; Vallejo et al., 2015), based on predicted values from the linear mixed models (ITT). Between-group differences in MCII pain coping were examined using χ2. Analyses were performed with IBM SPSS 28 and statistical significance was set at *p* < 0.05 (two-tailed). Protocol amendments are reported in Supplementary Appendix A. A data safety monitoring board was not used due to low anticipated risk.

## 3 Results

### 3.1 Sample characteristics

**Fig. 1:**
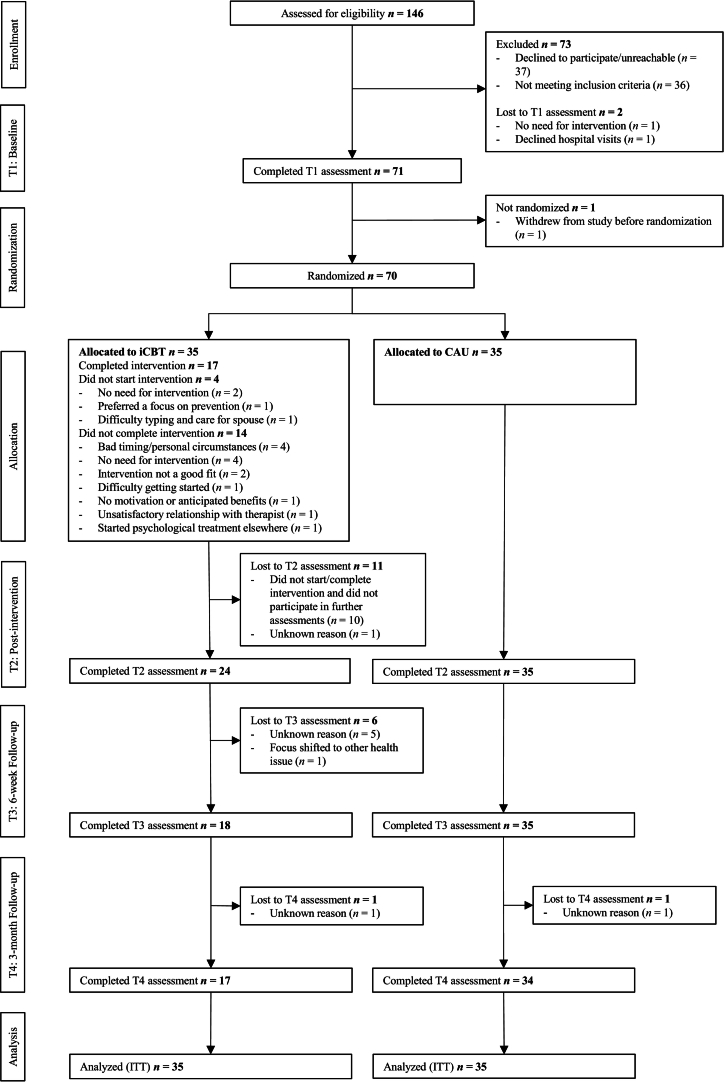
Flowchart hand osteoarthritis study. Note: iCBT = internet-based cognitive-behavioral therapy; CAU = care-as-usual; ITT = intention to treat.

Baseline characteristics are reported in Table S2 (hand osteoarthritis) and Table S3 (fibromyalgia, Supplementary Appendix D), and treatment exposure and therapist contact in Supplementary Appendix B, including Table S1. In the hand osteoarthritis study, 146 patients were assessed for eligibility between 20 June 2016 and 19 December 2019, with the last follow-up on 19 May 2021 (Fig. 1). Seventy patients (mean age 62.39 ± 7.58) were randomized to care-as-usual plus iCBT or care-as-usual only (both groups *n* = 35). Intervention completers and non-completers were similar at baseline, except for high blood pressure treatment, which was more common among intervention non-completers (8/18; 44.4%) than completers (1/17; 5.9%, *p* = 0.02). ICBT duration for completers ranged between 8 and 36 weeks (*M* = 17.9 ± 6.7) for completers, implying that some patients attained their goals in 8 weeks (minimum).

**Fig. 2:**
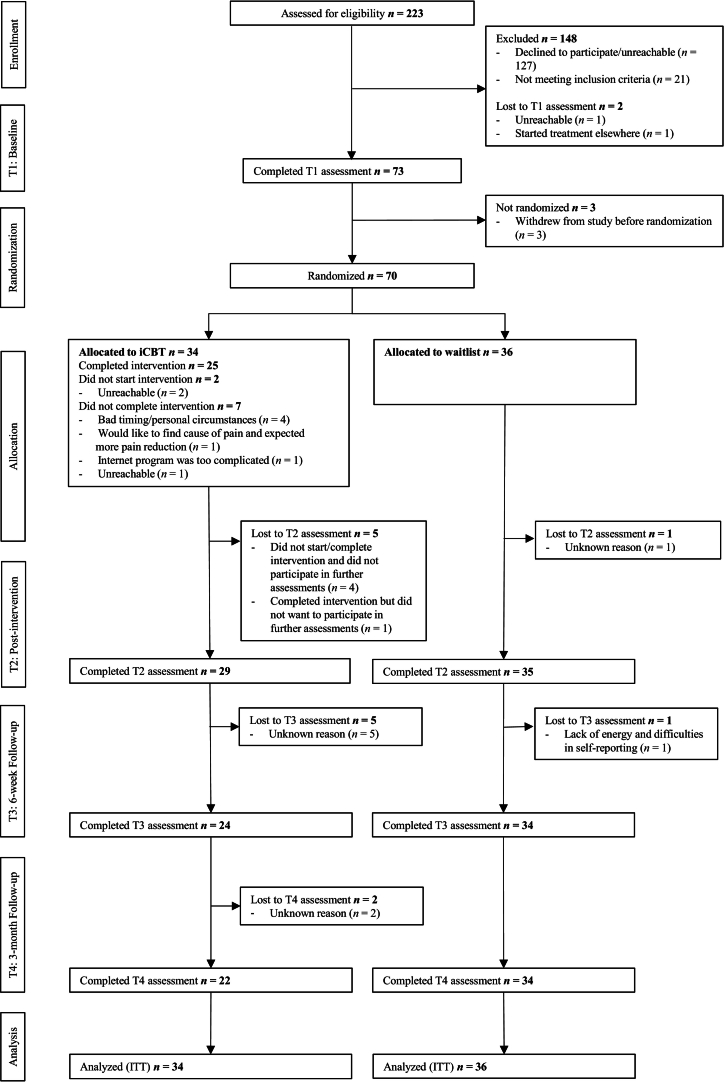
Flowchart fibromyalgia study. Note: Several patients left their contact information after reading concise information about the study online but afterwards were either not reachable or declined to participate after receiving more information, hence the ineligibility rates. iCBT = internet-based cognitive-behavioral therapy; ITT = intention to treat.

In the fibromyalgia study, 223 patients were assessed between 25 January 2017 and 3 December 2019, with the last follow-up on 26 January 2021 (Fig. 2). Seventy patients (mean age 46.43 ± 11.80) were randomized to iCBT (*n* = 34) or a waitlist (*n* = 36). Baseline characteristics did not differ between completers (*n* = 25) and non-completers (*n* = 9; *p* > 0.05). ICBT duration for completers ranged from 7 to 41 weeks (*M* = 20.6 ± 8.6).

Concomitant psychological treatment during the study was assessed only in the fibromyalgia study. Forty-four patients out of 70 (62.8%) reported no psychological treatment, 12 (17.1%) received some (3/34 [8.8%] in iCBT; 9/36 [25.0%] in control), and 14 (20.0%) had missing data. Reported reasons included depression, anxiety, and/or eating disorders. In the control group, eight out of 36 (22.2%) reported using medication for the symptoms they received additional treatment for, whereas none in the iCBT group did.

### 3.2 Primary and secondary outcomes

Descriptive statistics, change-score differences, and effect sizes with confidence intervals are in Table S4 (hand osteoarthritis) and Table S5 (fibromyalgia, Supplementary Appendix E, including assumption checks). Linear mixed model results are in Table 2 (hand osteoarthritis) and Table 3 (fibromyalgia), and intercepts in Tables S10 and S11 (Supplementary Appendix H).

**Table 2:** Results^a^ of linear mixed model analyses for primary and secondary outcomes in the hand osteoarthritis study.

	Short-term*group^b^ (T2 – T1)			Mid-term*group^b^ (T3 – T1)			Long-term*group^b^ (T4 – T1)		
	Est	*SE*	*p*	Est	*SE*	*p*	Est	*SE*	*p*
**Primary outcome**									
Pain coping (VAS)	0.64	0.48	0.187	1.09	0.52	**0.039**	0.37	0.53	0.488
**Secondary outcomes**									
*Psychological functioning*									
Well-being (VAS)	0.29	0.48	0.543	0.79	0.52	0.130	−0.37	0.53	0.492
RAND-36									
Mental Composite Score	5.48	3.74	0.144	2.83	4.04	0.485	6.84	4.12	0.098
Emotional Well-being	5.32	2.90	0.067	0.59	3.09	0.848	7.32	3.15	**0.021**
MPI-DLV									
Affective Distress	−0.21	0.27	0.423	0.42	0.29	0.141	−0.64	0.29	**0.030**
ICQ									
Helplessness	−0.29	0.60	0.628	−1.25	0.64	0.051	0.31	0.65	0.628
Acceptance	1.15	0.71	0.109	0.98	0.76	0.199	1.60	0.77	**0.040**
PCI									
Active Pain Coping	0.11	1.07	0.921	1.39	1.14	0.223	0.07	1.16	0.951
Transformation	−0.70	0.51	0.174	0.05	0.55	0.934	−0.50	0.56	0.372
Distraction	0.55	0.71	0.437	1.13	0.76	0.138	0.66	0.78	0.398
Reducing Demands	0.23	0.45	0.604	0.15	0.48	0.763	−0.17	0.49	0.730
Passive Pain Coping	−1.87	1.51	0.220	−1.21	1.61	0.455	−3.28	1.64	**0.047**
Retreating	−0.75	0.62	0.228	−1.00	0.66	0.141	−1.66	0.68	**0.015**
Worrying	−1.28	1.03	0.215	−0.55	1.10	0.616	−1.34	1.12	0.235
Resting	0.24	0.49	0.619	0.39	0.52	0.448	−0.21	0.53	0.690
IPQ-R									
Identity	−0.80	0.34	**0.020**	−0.81	0.37	**0.029**	−0.66	0.37	0.078
Timeline Acute/Chronic^c^	0.07	0.08	0.357	−0.15	0.09	0.080	0.06	0.09	0.507
Timeline Cyclical	−0.34	0.66	0.607	−0.76	0.70	0.279	−0.47	0.72	0.516
Consequences^d^	−0.01	0.02	0.616	−0.04	0.02	0.075	−0.03	0.02	0.171
Personal Control	−0.07	0.79	0.928	0.31	0.85	0.718	−0.29	0.87	0.739
Treatment Control	0.61	0.58	0.293	0.40	0.61	0.514	0.31	0.62	0.617
Emotional Rep	−0.64	0.95	0.502	−1.81	1.00	0.072	−2.98	1.02	**0.004**
Illness Coherence	−0.06	0.79	0.942	−0.15	0.83	0.857	0.15	0.85	0.863
MPI-DLV									
Life Control^c^	0.02	0.04	0.617	−0.02	0.05	0.600	0.16	0.05	**<0.001**
*Physical functioning*									
Pain (VAS)	−0.59	0.47	0.215	−0.80	0.51	0.117	−0.71	0.52	0.173
AUSCAN									
Pain	−0.23	0.67	0.734	−0.31	0.71	0.663	−0.37	0.72	0.607
Disability	−0.45	1.08	0.676	−0.40	1.15	0.727	−2.69	1.17	**0.022**
MPI-DLV									
Pain Severity	0.45	0.28	0.107	0.18	0.30	0.558	−0.35	0.30	0.247
RAND-36									
Phys. Composite Score	0.76	2.96	0.799	−1.71	3.19	0.593	4.15	3.24	0.202
Physical Functioning	−0.22	3.52	0.950	−0.90	3.79	0.813	2.97	3.86	0.443
Bodily Pain	5.82	3.56	0.103	1.24	3.84	0.747	2.34	3.92	0.550
Energy/Fatigue	−0.57	3.42	0.867	1.53	3.65	0.675	2.73	3.72	0.463
Health Change	8.76	5.07	0.085	5.53	5.50	0.316	14.95	5.61	**0.008**
*Impact on daily life*									
MPI-DLV									
Interference	0.07	0.20	0.735	−0.08	0.22	0.726	−0.27	0.22	0.221
Support^d^	0.06	0.03	0.108	0.02	0.04	0.657	−0.04	0.04	0.225
RAND-36									
Role Limitations due to Physical Health Problems	0.86	7.66	0.911	−6.97	8.25	0.399	8.38	8.41	0.320
General Health Perceptions	−0.55	3.06	0.859	2.39	3.26	0.464	5.59	3.32	0.094
Role Limitations due to Personal or Emotional Problems^c^	0.20	0.21	0.355	−0.11	0.23	0.642	0.32	0.24	0.179
Social Functioning^c^	0.20	0.16	0.224	0.20	0.18	0.270	0.04	0.18	0.834

Note: Bold face indicates a significant time-by-group interaction. Intercepts are reported in Supplementary Appendix H, Table S10. EST = the estimate of the regression coefficient (for short-term*group, mid-term*group, and long-term*group: the change in the variable from baseline for the intervention group compared to the control group); *SE* = standard error; VAS = visual analogue scale; RAND-36 = RAND 36-item Health Survey; MPI-DLV = Multidimensional Pain Inventory-Dutch Language Version; ICQ = Illness Cognition Questionnaire; PCI = Pain Coping Inventory; IPQ-R = Illness Perception Questionnaire-Revised; AUSCAN = Australian/Canadian Hand Osteoarthritis Index.

^a^Time (compared to baseline) as three dummy variables, age, and sex were included in all models, but coefficients are not reported.

^b^The models included fixed effects of time as three dummy variables: “short-term” (post-intervention vs. baseline), “mid-term” (6-week follow-up vs. baseline), and “long-term” (3-month follow-up vs. baseline). To examine iCBT effects over time, fixed effects of interactions between group (i.e., iCBT or CAU) and timepoint were included as three dummy variables: short-term*group, mid-term*group, and long-term*group.

^c,d^Due to non-normal distributions, these variables were transformed before using them in the linear mixed model analyses in the following way: ^c^inverse and log10-transformation (negatively skewed) and ^d^log10-transformation (positively skewed). Results of the transformed variables are displayed.

**Table 3:** Results^a^ of linear mixed model analyses for primary and secondary outcomes in the fibromyalgia study.

	Short-term*group^b^ (T2 – T1)			Mid-term*group^b^ (T3 – T1)			Long-term*group^b^ (T4 – T1)		
	Est	*SE*	*p*	Est	*SE*	*p*	Est	*SE*	*p*
**Primary outcome**									
Pain coping (VAS)	1.37	0.45	**0.003**	0.27	0.48	0.572	0.81	0.49	0.102
**Secondary outcomes**									
*Psychological functioning*									
Well-being (VAS)	0.96	0.41	**0.019**	0.94	0.43	**0.030**	0.97	0.44	**0.030**
RAND-36									
Mental Composite Score	2.98	4.14	0.473	5.48	4.33	0.207	3.25	4.42	0.464
Emotional Well-being	−0.71	3.36	0.833	1.09	3.48	0.754	−3.62	3.55	0.309
MPI-DLV									
Affective Distress	−0.31	0.26	0.245	−0.39	0.27	0.154	−0.40	0.28	0.154
ICQ									
Helplessness	−0.80	0.62	0.194	−0.83	0.63	0.191	−0.64	0.65	0.325
Acceptance	1.37	0.59	**0.020**	0.59	0.60	0.330	1.62	0.61	**0.009**
PCI									
Active Pain Coping	1.06	0.87	0.225	1.68	0.90	0.063	1.65	0.92	0.074
Transformation^c^	0.00^f^	0.03	0.893	0.07	0.03	**0.028**	0.03	0.03	0.287
Distraction	0.80	0.49	0.100	0.85	0.50	0.092	1.31	0.51	**0.011**
Reducing Demands	0.71	0.41	0.087	−0.11	0.43	0.794	0.24	0.44	0.583
Passive Pain Coping	−1.29	1.37	0.348	−0.21	1.41	. 882	−0.77	1.44	0.595
Retreating	0.72	0.59	0.225	0.93	0.61	0.128	2.01	0.62	**0.001**
Worrying	−2.17	0.92	**0.019**	−1.13	0.94	0.232	−2.35	0.96	**0.015**
Resting	0.14	0.54	0.789	−0.01	0.55	0.993	−0.41	0.56	0.471
IPQ-R									
Identity	0.67	0.46	0.148	0.02	0.47	0.972	−0.46	0.48	0.340
Timeline Acute/Chronic^d^	−0.03	0.07	0.681	−0.11	0.07	0.102	−0.02	0.07	0.798
Timeline Cyclical	−0.43	0.60	0.476	−0.14	0.62	0.822	−0.16	0.63	0.801
Consequences	−0.37	0.63	0.561	−0.86	0.65	0.182	−0.64	0.66	0.330
Personal Control	0.02	0.78	0.978	−0.61	0.80	0.449	0.19	0.82	0.815
Treatment Control	−0.42	0.57	0.456	−1.26	0.58	**0.032**	−0.47	0.60	0.432
Emotional Rep	−0.93	0.89	0.297	−0.71	0.91	0.442	−1.67	0.93	**0**.075
Illness Coherence	1.01	0.73	0.166	1.23	0.75	0.103	1.32	0.77	0.086
MPI-DLV									
Life Control	0.25	0.23	0.276	0.33	0.24	0.168	0.45	0.24	0.065
*Physical functioning*									
Pain (VAS)	−0.11	0.41	0.788	−0.38	0.44	0.388	−1.35	0.45	**0.003**
MPI-DLV									
Pain Severity	−0.09	0.28	0.752	−0.07	0.29	0.807	−0.30	0.30	0.305
RAND-36									
Physical Composite Score	5.81	3.05	0.058	4.44	3.18	0.164	10.51	3.24	**0.001**
Physical Functioning	3.66	3.37	0.278	2.67	3.51	0.447	4.50	3.58	0.210
Bodily Pain	5.61	4.03	0.166	2.81	4.18	0.503	11.94	4.28	**0.006**
Energy/Fatigue	5.99	3.44	0.083	7.58	3.62	**0.039**	6.93	3.64	0.058
Health Change	14.24	6.15	**0.021**	12.95	6.48	**0.047**	13.29	6.62	**0.046**
*Impact on daily life*									
MPI-DLV									
Interference	−0.11	0.19	0.539	−0.29	0.19	0.128	−0.65	0.20	**0.001**
Support	−0.20	0.26	0.446	0.28	0.27	0.291	−0.22	0.27	0.421
RAND-36									
Role Limitations due to Physical Health Problems	9.44	6.94	0.175	4.27	7.26	0.557	20.14	7.42	**0.007**
General Health Perceptions^e^	0.45	0.28	0.114	0.63	0.29	**0.033**	0.33	0.30	0.274
Role Limitations due to Personal or Emotional Problems	1.53	10.66	0.886	7.54	11.18	0.500	8.76	11.45	0.445
Social Functioning	10.93	4.53	**0.017**	10.92	4.68	**0.021**	5.51	4.78	0.251
FIQ	−4.16	2.91	0.154	−5.73	3.06	0.062	−6.39	3.12	**0.042**

Note: Bold face indicates a significant time-by-group interaction. Intercepts are reported in Supplementary Appendix H, Table S11. EST = the estimate of the regression coefficient (i.e., for short-term*group, mid-term*group, and long-term*group: the change in the variable from baseline for the intervention group compared to the control group); *SE* = standard error; VAS = visual analogue scale; RAND-36 = RAND 36-item Health Survey; MPI-DLV = Multidimensional Pain Inventory-Dutch Language Version; ICQ = Illness Cognition Questionnaire; PCI = Pain Coping Inventory; IPQ-R = Illness Perception Questionnaire-Revised; FIQ = Fibromyalgia Impact Questionnaire.

^a^Time (compared to baseline) as three dummy variables, age, and sex were included in all models, but coefficients are not reported.

^b^The models included fixed effects of time as three dummy variables: “short-term” (post-intervention vs. baseline), “mid-term” (6-week follow-up vs. baseline), and “long-term” (3-month follow-up vs. baseline). To examine iCBT effects over time, fixed effects of interactions between group (i.e., iCBT or CAU) and timepoint were included as three dummy variables: short-term*group, mid-term*group, and long-term*group.

^c,d,e^Due to non-normal distributions, these variables were transformed before using them in the linear mixed model analyses in the following way: ^c^log10-transformation (positively skewed); ^d^inverse and log10-transformation (negatively skewed); and ^e^square-root transformation (positively skewed). Results of the transformed variables are displayed.

^f^Estimates between −0.004 and 0.004 were rounded to 0.00.

#### 3.2.1 Hand osteoarthritis: primary outcome

**Fig. 3:**
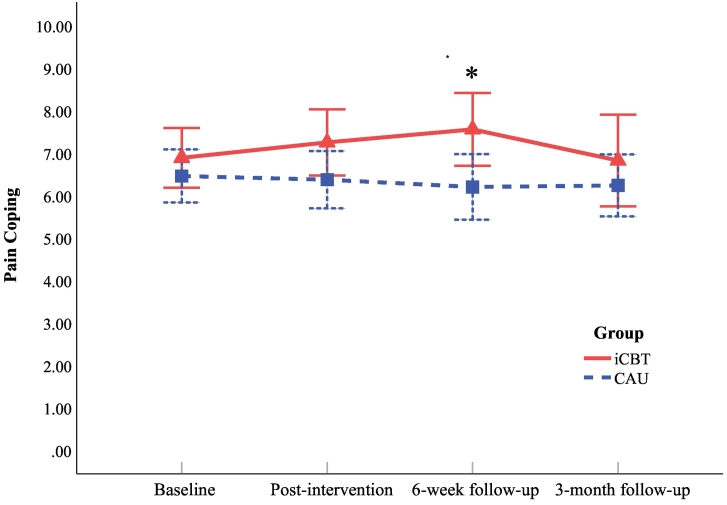
Hand osteoarthritis: observed mean pain coping scores at baseline, post-intervention, 6-week follow-up, and 3-month follow-up for the iCBT group and the CAU group. Note: Higher pain coping scores indicate better pain coping. Time from baseline to post-intervention varied between participants; iCBT = internet-based cognitive-behavioral therapy; CAU = care-as-usual. * = participants in the iCBT group improved significantly more in pain coping (compared to baseline) than participants in the CAU group at 6-week follow-up (*p* = 0.039), tested with linear mixed models, see Table 2.

In hand osteoarthritis, no differences between the intervention and control group were found in pain coping at post-intervention (*p* = 0.187; Fig. 3) and 3-month follow-up (*p* = 0.488). However, patients in the intervention group improved significantly more in pain coping at 6-week follow-up than controls (*p* = 0.039; *d* = 0.41). Of the intervention group, 14/35 (40.0%) showed a minimal clinically important improvement of 10% in pain coping, which did not differ significantly from the control group (10/35 [28.6%]; *p* = 0.314).

#### 3.2.2 Fibromyalgia: primary outcome

**Fig. 4:**
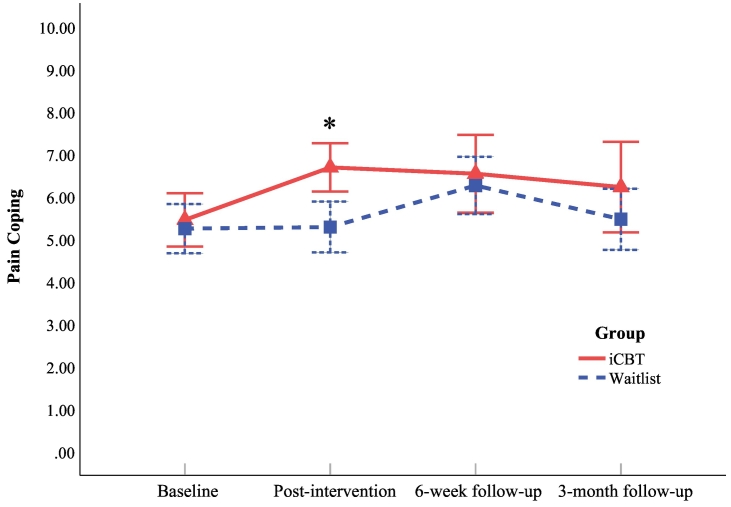
Fibromyalgia: observed mean pain coping scores at baseline, post-intervention, 6-week follow-up, and 3-month follow-up for the iCBT group and the waitlist group. Note: Higher pain coping scores indicate better pain coping. Time from baseline to post-intervention varied between participants; iCBT = internet-based cognitive-behavioral therapy. * = participants in the iCBT group improved significantly more in pain coping (compared to baseline) than participants in the waitlist group at post-intervention (*p* = 0.003), tested with linear mixed models, see Table 3.

In fibromyalgia, patients in the intervention group improved significantly more in pain coping at post-intervention (*p* = 0.003; *d* = 0.60) than controls (Fig. 4). No significant time-by-group interactions were found for pain coping for any of the other time points (*p* ≥ 0.102). Of the intervention group, 23/33 (69.7%) showed a minimal clinically important improvement of 10% in pain coping, which differed significantly from the control group (14/36 [38.9%]; *p* = 0.010). One patient in the intervention group had missing values on VAS pain coping at both time points and could not be included in the MCII calculation.

#### 3.2.3 Hand osteoarthritis and fibromyalgia: secondary outcomes

##### 3.2.3.1 Psychological outcomes: well-being

In hand osteoarthritis, significant time-by-group interactions were found at *3-month follow-up* for Emotional Well-being (RAND-36; *p* = 0.021; *d* = 0.58) and Affective Distress (MPI-DLV; *p* = 0.030; *d* = 0.81), with patients in the intervention group reporting higher well-being and less distress than controls (Table 2). In fibromyalgia, at all time points, significant interactions were found for Well-being (VAS; *p* ≤ 0.030; 0.30 ≤ *d* ≤ 0.34), with patients in the intervention group reporting higher well-being than controls (Table 3).

##### 3.2.3.2 Psychological outcomes: coping and cognitions

In hand osteoarthritis, at *post-intervention* and *6-week follow-up*, significant interactions were found for Identity (IPQ-R; *p* ≤ 0.029; 0.48 ≤ *d* ≤ 0.50), with patients in the intervention group attributing fewer symptoms to hand osteoarthritis than controls. Moreover, at *3-month follow-up*, significant interactions were found in hand osteoarthritis for Acceptance (ICQ; *p* = 0.040; *d* = 0.54), Passive Pain Coping (PCI; *p* = 0.047; *d* = 0.65), Retreating (PCI; *p* = 0.015; *d* = 0.82), Emotional Representations (IPQ-R; *p* = 0.004; *d* = 0.76), and Life Control (MPI-DLV; *p* < 0.001; *d* = 0.98), with patients in the intervention group reporting higher illness acceptance, less passive pain coping, less retreating, less negative emotions regarding the illness, and higher life control than controls (Table 2).

In fibromyalgia, at *post-intervention*, significant interactions were found for Acceptance (ICQ; *p* = 0.020; *d* = 0.45) and Worrying (PCI; *p* = 0.019; *d* = 0.53), with patients in the intervention group reporting higher illness acceptance and less worrying about their pain than controls. At *6-week follow-up*, significant interactions were found in fibromyalgia for Transformation (PCI; *p* = 0.028; *d* = 0.49) and Treatment Control (IPQ-R; *p* = 0.032; *d* = −0.63), with patients in the intervention group reporting more pain transformation as a coping strategy but less positive beliefs about treatment effectiveness than controls. Finally, at *3-month follow-up*, significant interactions were found in fibromyalgia for Acceptance (ICQ; *p* = 0.009; *d* = 0.50), Distraction (PCI; *p* = 0.011; *d* = 0.73), Retreating (PCI; *p* = 0.001; *d* = −0.95), and Worrying (PCI; *p* = 0.015; *d* = 0.59), with patients in the intervention group reporting higher illness acceptance, more distraction from pain, more retreating, and less worrying about their pain than controls (Table 3).

##### 3.2.3.3 Physical outcomes

In hand osteoarthritis, at *3-month follow-up*, significant interactions were found for Disability (AUSCAN; *p* = 0.022; *d* = 0.65) and Health Change (RAND-36; *p* = 0.008; *d* = 0.57), with patients in the intervention group reporting less disability and a larger improvement in perceived health than controls (Table 2). In fibromyalgia, at all time points, significant interactions were found for Health Change (RAND-36; *p* ≤ 0.047; 0.53 ≤ *d* ≤ 0.66), with patients in the intervention group reporting a larger improvement in perceived health than controls. At *6-week follow-up*, a significant interaction was found in fibromyalgia for Energy/Fatigue (RAND-36; *p* = 0.039; *d* = 0.59), with patients in the intervention group reporting having more energy than controls. At *3-month follow-up*, significant interactions were found in fibromyalgia for Pain (VAS; *p* = 0.003; *d* = 0.63), Bodily Pain (RAND-36; *p* = 0.006; *d* = 0.57) and Physical Composite Score (RAND-36; *p* = 0.001; *d* = 0.61), with patients in the intervention group reporting less pain and better physical functioning than controls (Table 3).

##### 3.2.3.4 Impact on daily life outcomes

In hand osteoarthritis, no significant interactions were found at any of the time points for impact on daily life outcomes (Table 2). Regarding impact on daily life outcomes in fibromyalgia, at *post-intervention* and *6-week follow-up*, significant interactions were found for Social Functioning (RAND-36; *p* ≤ 0.021; 0.52 ≤ *d* ≤ 0.53), with patients in the intervention group reporting less interference in social activities due to emotional problems or physical health than controls. Furthermore, at *6-week follow-up*, a significant interaction was found in fibromyalgia for General Health Perceptions (RAND-36; *p* = 0.033; *d* = 0.43), with patients in the intervention group reporting more positive health perceptions than controls. Finally, at *3-month follow-up*, significant interactions were found in fibromyalgia for Role Limitations due to Physical Problems (RAND-36; *p* = 0.007; *d* = 0.46), Interference (MPI-DLV; *p* = 0.001; *d* = 0.63), and FIQ (*p* = 0.042; *d* = 0.37), with patients in the intervention group reporting less role limitations due to physical health, less pain interference, and less fibromyalgia impact than controls (Table 3).

### 3.3 Module completion, treatment evaluation, and adverse events

In the hand osteoarthritis study, of the completers, two (11.8%) worked on one module, two (11.8%) worked on two modules, three (17.6%) worked on three modules, five (29.4%) worked on four modules, and four (23.5%) worked on five modules (module completion data of one patient was missing). In the fibromyalgia study, of the completers, one (4.0%) worked on two modules, five (20.0%) worked on three modules, one (4.0%) worked on four modules, two (8.0%) worked on five modules, and 16 (64.0%) worked on six modules. Patients were sufficiently satisfied with the therapeutic relationship and the intervention (Supplementary Appendix F, including Tables S6 and S7). The intervention received a mean score of 6.95 (*SD* = 1.88) and 6.92 (*SD* = 1.98) out of 10 in the hand osteoarthritis study and fibromyalgia study, respectively. After randomization, one patient in the hand osteoarthritis control group reported a non-trial-related adverse event (diagnosis of several medical conditions) and continued participating. No adverse events were reported in the fibromyalgia study.

### 3.4 Sensitivity analyses

In both studies, results were largely consistent between analyses with and without covariates, with no meaningful differences in the size, direction, or significance of time-by-group interactions. Regarding the completers analysis, in hand osteoarthritis, the primary outcome pain coping showed a stronger effect at 6 weeks in the completers analysis with covariates than in the primary analysis. A secondary outcome (Health Change [RAND-36]) reached statistical significance only in completers post-intervention, while others (e.g., Passive Pain Coping [PCI] at 3-month follow-up) lost statistical significance. In fibromyalgia, the primary outcome showed a slightly weaker effect post-intervention among completers, while some secondary outcomes (e.g., Energy/Fatigue [RAND-36] at 3 months) reached statistical significance only in completers. A few other outcomes (e.g., Well-being [VAS] at 6 weeks) lost statistical significance. Most other effects were consistent across analyses and excluding covariates had minimal impact (Supplementary Appendix G, including Tables S8 and S9).

## 4 Discussion

These studies evaluated the effectiveness of one guided iCBT for patients with hand osteoarthritis or fibromyalgia, recruited in routine care, with longer term follow-up. In hand osteoarthritis, the primary hypothesis was not supported, showing no significant between-group differences in pain coping at post-intervention. However, at 6-week follow-up, patients in the intervention group reported better pain coping than controls. In fibromyalgia, the primary hypothesis was supported, showing a medium to large improvement in pain coping in the intervention group compared to the control group at post-intervention, although results were not sustained. More patients in the fibromyalgia intervention group than the control group also showed a minimal clinically important improvement of 10% in pain coping at post-intervention. Moreover, both studies found significant secondary outcome effects, mainly at 3-month follow-up. Patients in the hand osteoarthritis intervention group showed higher well-being, more adaptive and less maladaptive illness cognitions, less maladaptive pain coping strategies, and better physical functioning than controls, with medium to large effects. However, no significant effects were found for hand osteoarthritis impact outcomes. Patients in the fibromyalgia intervention group showed higher well-being, more adaptive illness cognitions, more adaptive and less maladaptive coping strategies, better physical functioning, and a lesser disease impact than controls, with small to large effects. Secondary outcome findings are exploratory and should be interpreted cautiously due to a lack of statistical correction.

The findings on coping and cognitions in fibromyalgia support and extend previous iCBT research in fibromyalgia (Terpstra et al., 2021; Vallejo et al., 2015) and chronic pain (Gandy et al., 2022; Terpstra et al., 2022a). Whereas earlier work identified relaxation as the only effective coping strategy in fibromyalgia (Vallejo et al., 2015), we observed improvements in multiple strategies, including pain transformation and distraction. The absence of sustained effects on VAS pain coping in fibromyalgia may be explained by improvements in the waitlist group at 6-week follow-up.

The findings on coping and cognitions are new for hand osteoarthritis, with no prior iCBT trials in this group, and partially align with previous research in other osteoarthritis populations (Zhang et al., 2018). While a meta-analysis on psychological interventions in osteoarthritis reported large post-intervention improvements in pain coping scales (mean differences = 1.64–22.21, *p* ≤ 0.05) in favor of a psychological intervention compared to a passive control (Zhang et al., 2018), we found a small to medium effect for pain coping at 6-week follow-up. Rather high baseline coping scores may have limited improvement due to a ceiling effect (Arslan and Benke, 2023).

Improvements in well-being outcomes in hand osteoarthritis and fibromyalgia align with prior research in fibromyalgia and arthritis showing mostly positive effects of guided iCBT on depression (*d* = 0.54–0.87) and anxiety (*d* = 0.48–0.67) compared to passive controls (Terpstra et al., 2021). The effects on physical and disease impact outcomes in fibromyalgia are also consistent with previous findings (Terpstra et al., 2021). In hand osteoarthritis, improvements in physical functioning and the lack of effects on disease impact largely mirror a systematic review of digital self-management programs for osteoarthritis, which found effects on physical function (SMD = −0.26), pain (SMD = −0.28), and disability (SMD = −0.10), and no effects on quality of life compared to passive controls (Safari et al., 2020). In our study, the absence of pain effects (as opposed to Safari et al., 2020) and disease impact effects (consistent with Safari et al., 2020) may reflect favorable baseline scores, leaving little room for improvement.

In line with our baseline scores, another previous study suggests that HR-QoL is higher in hand osteoarthritis than in fibromyalgia, with the former being close to those of healthy controls (Salaffi et al., 2019). Moreover, a different study found that hand osteoarthritis was associated with reduced physical, but not mental HR-QoL in a general population and patients in hospital care (Loef et al., 2020). The impact of hand osteoarthritis may be generally lower than that of fibromyalgia. Supporting this, in our studies, patients with hand osteoarthritis had neuroticism scores comparable to those of a general population, whereas patients with fibromyalgia appeared to have higher scores than both a general population and patients with cancer or heart conditions (Sanderman et al., 2012). High neuroticism has previously been associated with the development of somatic and mental disorders and pain vigilance (Naylor et al., 2017). Additionally, non-completion was more frequent in the hand osteoarthritis study than the fibromyalgia study, partly because non-completers perceived little need for the intervention or expected no benefit, potentially reflecting a lower disease burden.

In both hand osteoarthritis and fibromyalgia, most effects emerged at 3-month follow-up, possibly reflecting the time and practice needed to apply new coping strategies. While long-term between-group follow-up studies on guided iCBT for chronic pain are limited (Terpstra et al., 2022a), some evidence supports sustained effects in rheumatic and musculoskeletal diseases (Terpstra et al., 2021). Delayed effects may also relate to continued access to intervention content post-intervention (Vallejo et al., 2015).

Future research could explore booster sessions to sustain intervention effects, as they have shown benefits in mental health conditions, such as obsessive-compulsive disorder (Andersson et al., 2014). However, evidence for booster effects in musculoskeletal conditions is limited and low quality, and delivery method (remote versus face-to-face) did not influence effects (Buzasi et al., 2022). A high-quality trial testing theory-based boosters with varying frequency and duration is needed in rheumatic and musculoskeletal diseases.

### 4.1 Strengths and limitations

Strengths of the studies include the overarching approach in evaluating iCBT across two RCTs in different chronic pain conditions, covering a wide range of outcomes. This was also the first evaluation of therapist-guided iCBT in hand osteoarthritis, highlighting its potential for improving self-management. A further strength is the flexible treatment duration, allowing therapists to tailor the number of treatment weeks to patients' individual needs and progress. This adaptability may enhance the ecological validity and clinical applicability of the iCBT.

Some limitations should be considered as well. The power analysis was based on a repeated-measures ANOVA, but similar variance components and equal or superior power of linear mixed models (Gueorguieva and Krystal, 2004; Ma et al., 2012) indicate that the study was adequately powered relative to the original calculation. Drop-out was substantial in both iCBT groups and was not accounted for in the sample size calculation due to feasibility constraints. This may have reduced power, although linear mixed models handle missing data well under the Missing At Random Assumption (MAR) (Gabrio et al., 2022; Peters et al., 2012) and ITT analyses provide conservative estimates of treatment effects. Differential attrition, especially in the hand osteoarthritis study, may challenge the MAR assumption. However, baseline comparisons and sensitivity analyses using completers-only datasets generally support the robustness of our findings. Drop-out is common in iCBT studies (Terpstra et al., 2022a), while more personal therapist–patient contact could potentially improve retention (Leterme et al., 2020).

Furthermore, although multilevel modeling accounts for data dependencies, it does not eliminate the risk of an inflated familywise error rate due to multiple testing of secondary outcomes (Rubin, 2024). Consequently, statistically significant effects observed for individual secondary outcomes should be interpreted with caution and viewed as exploratory rather than confirmatory. Greater weight should be placed on the consistency and direction of effects across related outcomes and time points, rather than on isolated significant findings. Also, transformations did not improve the distribution of Physical Functioning (RAND-36) in the hand osteoarthritis study, so these results should be interpreted cautiously.

Next, experiencing a minimum pain level was not an inclusion criterion. Two patients in the fibromyalgia intervention group and eleven in the hand osteoarthritis intervention group reported little pain (score 1, 2 or 3 on VAS pain) at baseline; these patients may have benefitted less from the intervention. Moreover, in the fibromyalgia study, inclusion was based on previous fibromyalgia diagnoses. It was unclear which diagnostic criteria were used as criteria have been amended over the years (Wolfe et al., 2016), which could have resulted in a somewhat heterogeneous sample. Besides, psychiatric comorbidity rates at baseline were relatively low (<6% in either group in the hand osteoarthritis study and <20% in either group in the fibromyalgia study) compared with previous reports. For example, lifetime depression in fibromyalgia can reach 63%, with about one-third of patients presenting with bipolar disorder, panic disorder, or post-traumatic stress disorder (Kleykamp et al., 2021), while anxiety and depression in older adults (65–85 years) with hand osteoarthritis range from 9% to 31% (Siviero et al., 2016). Our baseline comorbidity rates likely reflect exclusion of participants with severe psychiatric disorders (e.g., borderline personality disorder, alcohol use disorder) or ongoing psychological treatment, to optimize patient safety, intervention fidelity, and a clear interpretation of outcomes. However, some fibromyalgia participants received additional psychological treatment during the study, particularly in the control group, suggesting that comorbidity may have been higher than baseline self-report indicated. Differential rates of such treatment could have reduced between-group differences. Low baseline comorbidity also limits generalizability, especially for fibromyalgia. Future studies should examine iCBT feasibility and effectiveness in more clinically complex populations. Additionally, race and ethnicity data were not collected, limiting conclusions about intervention equity. Finally, comparisons between the hand osteoarthritis and fibromyalgia studies should be made cautiously considering the different control groups and lack of statistical testing between patient populations.

### 4.2 Conclusion and future directions

In summary, guided iCBT improved the primary outcome of pain coping at the primary endpoint in fibromyalgia, whereas findings for hand osteoarthritis showed no superiority on the primary outcome at the primary endpoint (negative trial). Both trials suggested potential benefits on several secondary outcomes with medium to large effects for hand osteoarthritis and small to large effects for fibromyalgia, particularly at 3-month follow-up. However, these results need to be interpreted with caution, considering the exploratory nature of the secondary outcomes. For fibromyalgia, further research is required to confirm these effects and to determine whether, contingent on replication, guided iCBT could play a role within interdisciplinary care. A screening for disease impact at the start of a fibromyalgia care program and subsequent referral to a psychologist for iCBT in case of a high disease impact may improve intervention fit in a research context. In hand osteoarthritis, more research is also needed to clarify whether the exploratory secondary benefits can be reproduced. Future studies on guided iCBT should explore booster sessions, blended CBT formats to reduce drop-out, cost-effectiveness, and feasibility and effectiveness for a broad range of potential participants, including people with a low socioeconomic position and people from diverse racial and ethnic groups.

## CRediT authorship contribution statement

Authors made substantial contributions to the conception of the work (RvdV, MK, AE), data acquisition (JT, RvdV, MK, AE), analysis (JT, SvB, RvE, ED), and interpretation (all authors), drafting (JT, RvE) and revising the paper (all authors). All authors approved of the final version and agreed to be accountable for all aspects of the work. There is no one else who fulfils authorship criteria that has been excluded as an author.

## Funding sources

The hand osteoarthritis study was supported by 10.13039/501100001717Leiden University and 10.13039/501100005039Leiden University Medical Center, Leiden, the Netherlands: Programma Vernieuwing Zorg. The fibromyalgia study was supported by 10.13039/501100001717Leiden University and 10.13039/501100009248Innovatiefonds Zorgverzekeraars (2619). The funders had no role in the study design, data collection, analysis, and data interpretation, in writing the paper, or the decision to submit the paper.

## Declaration of competing interest

The authors declare that they have no known competing financial interests or personal relationships that could have appeared to influence the work reported in this paper.

## Data Availability

De-identified restructured data sets for the multilevel analyses, including additional documents (e.g., study protocols, statistical analysis plans, informed consent forms, questionnaires, SPSS syntax files, and data dictionaries) are available in a public, open access repository, namely, DataverseNL (data are available upon paper publication at: https://doi.org/10.34894/T9T4VR) under a Creative Commons Attribution Non-Commercial (CC-BY-NC 4.0) license. De-identified raw data are available upon reasonable request from DataverseNL under a CC-BY-NC 4.0 license.

## Appendix A Supplementary data

Supplementary material Image 1
