# Supplementary material for: An internet-based cognitive-behavioral self-management intervention for patients with hand osteoarthritis or fibromyalgia – Two randomized controlled trials
Source: Internet Interv. 2026 Jan 19;43:100908. doi: 10.1016/j.invent.2026.100908 (PMC12861268; doi:10.1016/j.invent.2026.100908)
Supplement: Supplementary file 1 — Supplementary material [file mmc1.docx]

**Supplementary Appendix A. Amendments to the hand osteoarthritis and fibromyalgia studies**

**Important amendments to methods after trial commencement**

All amendments to statistical methods in the protocols were approved by the medical ethics committee. In both studies, repeated-measures ANOVA was replaced by linear mixed modelling, since the latter is a more advanced statistical method that is better suited to deal with missing data than the former. Also, a within-group measure of minimal clinically important improvement was added, which replaced a previously described within-group Cohen’s *d*, that was of little value since no comparison with a control group would be made and its clinical relevance would be unclear. Furthermore, two-sided testing instead of one-sided testing was added to investigate changes in favor of the intervention as well as the control group. Lastly, the Bonferroni correction was removed. Multilevel models account for the dependence among repeated measures; applying a Bonferroni adjustment across all outcomes and time points would have been overly conservative and could increase the risk of Type II errors (Perneger, 1998). Exploratory correlation analyses were removed from the protocol because of insufficient power due to a limited sample size.

**Supplementary Appendix B. ICBT description, therapist guidance, and treatment exposure in the hand osteoarthritis and fibromyalgia studies**

**ICBT: intervention content**

The iCBT “Master Your Pain” was hosted by web-based platform “Karify” (uleiden.karify.com) and was offered to patients free of charge. The intervention started with one or two face-to-face intake sessions (1–1.5 hours in total) with a psychologist to familiarize the patient with the therapist and the intervention and to develop personal intervention goals. The iCBT consisted of six text-based modules: an introductory module including goal setting, modules on negative mood, activities, thoughts, and the social environment, and a final module covering relapse prevention and long-term goal setting. An overview of the homepage of the iCBT is shown in Figure S1 in Supplementary Appendix B. Each module contained several CBT components including pain education, practical assignments, relaxation training, and self-monitoring. The modules were aimed at learning how to cope with the consequences of a chronic condition in daily life. No changes were made in the functionality or content of the intervention during the trials. Based on the personal goals of the patient, the modules, the assignments within the modules, and the therapist’s feedback were tailored to meet the patient’s specific needs.

**Figure S1**

**Screenshot of the homepage of “Master Your Pain” showing all treatment modules.**


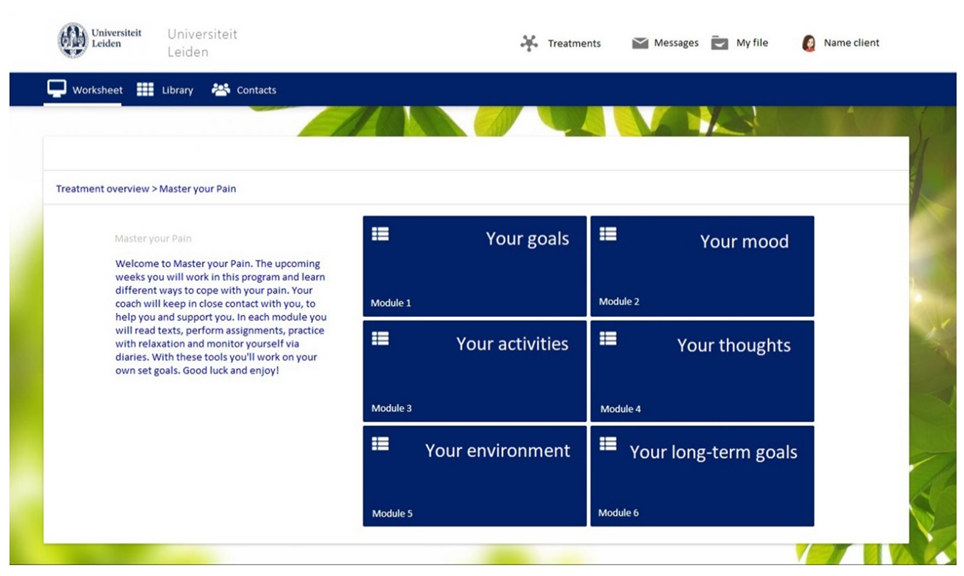


**Note:** Reprinted from Internet Interventions, 18, van der Vaart R, Worm-Smeitink M, Bos Y, Wensing M, Evers A, Knoop H, Implementing guided ICBT for chronic pain and fatigue: a qualitative evaluation among therapists and managers, Appendix B, Copyright Elsevier (2019).

**ICBT: therapist guidance**

Patients followed the web-based program at home and received asynchronous feedback from the psychologist via a secure online mailbox within the online program at least once a week. A reminder mail was sent by the therapist if patients had not sent in their weekly assignments. If no reaction was received, the therapist would call the patient to assess any difficulties in completing the assignments. Therapists spent an estimated 30 minutes on feedback per participant per week. Upon completion of the program, the psychologist provided two optional booster sessions via telephone (~15–30 minutes). In these sessions, strategies to further attain treatment goals and improve the achieved results were discussed. These sessions took place 1 month and 2,5 months after finishing the intervention. In the hand osteoarthritis study, the intervention was provided by two female psychologists with a master’s degree in health and medical psychology (age: 37 and 54 years; therapeutic experience: 11 and 2 years, respectively). In the fibromyalgia study, the intervention was provided by a male and a female psychologist with a master’s degree in clinical and health psychology and health and medical psychology, respectively (age: 38 and 54 years; therapeutic experience: 12 and 2 years, respectively). All psychologists received training in the use of the iCBT. A senior clinical psychologist with post-academic CBT training supervised the therapists.

**Treatment exposure and therapist contact**

Patients often worked on several modules at the same time. Among intervention completers, most time was spent on the thoughts module in the hand osteoarthritis study and on the activities module in the fibromyalgia study (further details are reported in Table S1 below). In the hand osteoarthritis study, therapists sent on average 12.86 messages to patients who started treatment after the intake (*SD* = 7.87; range 2-28) and patients sent on average 11.00 messages (*SD* = 9.64; range 0-33) to the therapists. In the fibromyalgia study, therapists sent on average 17.57 messages to patients (*SD* = 10.36; range 2-45) and patients sent on average 13.84 messages (*SD* = 9.34; range 0-34) to the therapists. On average, therapists called patients in the intervention group 1.50 times (*SD* = 1.56; range 0-6) in the hand osteoarthritis study and 2.13 times (*SD* = 1.26; range 0-5) in the fibromyalgia study. Common topics of the calls concerned providing additional instructions, promoting adherence, discussing terminating the program, and discussing personal or family circumstances.

**Table S1**

**Overview of treatment exposure of the intervention completers (*n* = 17) in the hand osteoarthritis study and the intervention completers (*n* = 25) in the fibromyalgia study**

| Module | Patients that worked  on the module  *n* (%) | |  |  | Weeks spent  on the module  *M* (*SD*; range) | |
| --- | --- | --- | --- | --- | --- | --- |
|  | Hand osteoarthritis | Fibromyalgia |  |  | Hand osteoarthritis | Fibromyalgia |
| Thoughts | 10 (58.8) | 21 (84.0) |  |  | 6.38 (1.80; 3-9) | 3.24 (1.87; 1-7) |
| Activities | 13 (76.5) | 23 (92.0) |  |  | 5.13 (3.65; 1-14) | 4.26 (3.11; 1-12) |
| Social environment | 5 (29.4) | 17 (68.0) |  |  | 3.35 (3.26; 1-9) | 2.88 (2.60; 1-10) |
| Long-term goals | 10 (58.8) | 19 (76.0) |  |  | 3.11 (2.80; 1-9) | 1.42 (0.90; 1-4) |
| Mood | 8 (47.1) | 22 (88.0) |  |  | 3.22 (2.41; 1-9) | 3.09 (2.94; 1-14) |
| Goals | 10 (58.8)^a^ | 25 (100.0) |  |  | 1.00 (0.00) | 1.48 (1.45; 1-8) |

**Note:** *n* = number; *M* = mean; *SD* = standard deviation.

^a^While all intervention completers set goals for the treatment during the intake and subsequently worked on them in the online environment, not all patients filled out these goals in the goal module.

**Supplementary Appendix C. Secondary outcomes, descriptive characteristics, and adverse events in the hand osteoarthritis and fibromyalgia studies**

The measures outlined below were used in both the hand osteoarthritis study and the fibromyalgia study, unless mentioned otherwise. A **visual analogue scale** (VAS; 0-10) was used to assess pain coping, with higher scores indicating better pain coping. VAS has shown good psychometric properties in a rheumatoid arthritis sample undergoing routine clinical care (Sendlbeck et al., 2015). In this sample, test–retest reliability of VAS pain was good across three time points over a follow-up period of up to 13 days (*r* = 0.82–0.95). Convergent validity with numerical and verbal rating scales was good (*r* = 0.82–0.92), and discriminant validity was supported by weak correlations with age (*r* = 0.01–0.12). Internal responsiveness across the three time points was low (standardized response means = 0.14–0.21), likely due to the short follow-up period. Although there is limited research on VAS specifically for pain *coping*, there is a precedent for using VAS to measure coping in other chronic somatic conditions. Raj-Koziak et al. (2018) validated a VAS for tinnitus coping in a clinical population, reporting acceptable test-retest reliability in two measurements over three days (ICC = 0.67) and moderate correlations with a global tinnitus index (*ρ* = 0.52).

The **Pain Coping Inventory** (PCI) is a self-reported 33-item questionnaire that assesses cognitive and behavioral pain coping strategies (Kraaimaat et al., 1997). The questionnaire consists of six scales; Pain Transformation, Distraction, and Reducing Demands are characterized as active pain coping strategies, and Retreating, Worrying, and Resting are classified as passive pain coping strategies. Items are scored on a 4-point Likert scale and summed to obtain scale scores, with higher scores indicating greater use of a coping strategy. The PCI has shown to be sufficiently valid and reliable, with Cronbach’s alphas ranging from .62 to .79 for separate subscales in different chronic pain samples (Kraaimaat et al., 1997).

Well-being was assessed with a **visual analogue scale** (VAS) ranging from 0 to 10, with higher scores indicating better well-being. Two subscales of the **Illness Cognition Questionnaire** (ICQ) were used, namely Helplessness (6 items) and Acceptance (6 items) (Evers et al., 2001). Items are scored on a 4-point Likert scale ranging from “not at all” to “completely”. Higher scale scores indicate greater use of the measured construct. The reliability and validity of the ICQ have been demonstrated across different samples of patients with chronic somatic conditions, with Cronbach’s alphas ranging from .84 to .90 for separate subscales in a sample of patients with rheumatoid arthritis (Evers et al., 2001).

The **Illness Perception Questionnaire-Revised** (IPQ-R) was used to assess several components of illness representations (Moss-Morris et al., 2002). The Identity Scale consists of yes/no-questions, whereas the Timeline Acute/Chronic, Timeline Cyclical, Consequences, Illness Coherence, Personal Control, Treatment Control, and Emotional Representations scales of the IPQ-R are rated on a 5-point Likert type scale ranging from “strongly disagree” to “strongly agree”. High scores on the Identity, Timeline Acute/Chronic and Cyclical, Consequences, and Emotional Representations scales reflect beliefs about the number of symptoms attributed to the illness, the chronicity and cyclical nature, and the negative consequences of the illness, and patients' emotional experience of their illness. High scores on the Personal and Treatment Control and Coherence scales reflect positive beliefs about perceived control and an understanding of the illness. The IPQ-R has shown to be valid and reliable, with Cronbach’s alphas ranging from .79 to .89 for separate subscales (Moss-Morris et al., 2002).

A **VAS** from 0 to 10 was used to assess pain, with higher scores indicating more severe pain. The **Multidimensional Pain Inventory-Dutch Language Version** (MPI-DLV) is a self-reported questionnaire that quantifies pain and behavior associated to pain (Lousberg et al., 1999), For this study, part 1 of the MPI-DLV containing 28 items was used, with the scales Interference and Pain Severity, and as exploratory measures Support, Life Control, and Affective Distress. Items are rated on various 7-point Likert scales, with higher scores indicating greater intensity in a subscale. The MPI-DLV has shown to be valid and reliable, with Cronbach’s alphas ranging from .63 to .89 in a sample of chronic pain patients (Lousberg et al., 1999).

The **RAND 36-item Health Survey** measures HR-QoL and comprises 36 items that are averaged together to provide eight health scale scores, namely Physical Functioning, Role Limitations due to Physical Health Problems, Role Limitations due to Personal or Emotional Problems, Social Functioning, Emotional Well-being, Energy/Fatigue, Bodily Pain, and General Health Perceptions (Hays et al., 1993). It also includes one item on perceived change in health. Moreover, unweighted physical and mental health composite scores were calculated (Andersen et al., 2022). Higher scores indicate more advantageous health states. The RAND-36 (Dutch version) showed sound psychometric properties overall in a sample of three chronic diseases, with reliability coefficients for the different scales ranging from .81 to .92 in a sample of patients with rheumatism (Moorer et al., 2001).

*Hand osteoarthritis only*: the Pain (5 items) and Disability (9 items) scales of the self-reported **Australian/Canadian Hand Osteoarthritis Index** (AUSCAN) were used to assess pain and disability (Bellamy et al., 2002). Items are rated on 5-point Likert scale ranging from “none” to “extreme”, with higher scores indicating more pain and disability. The AUSCAN has shown to be valid and reliable with Cronbach’s alphas ranging from .90 to .98 for separate subscales (Bellamy et al., 2002).

*Fibromyalgia only*: the **Fibromyalgia Impact Questionnaire** (FIQ) is a self-reported 10-item measure of the impact of fibromyalgia on various aspects of functioning and well-being (Burckhardt et al., 1991). Total scores range from 0 to 100, with higher scores indicating worse fibromyalgia impact. The FIQ has shown to have sound psychometric values, with Cronbach’s alphas of .81 for the sub-questions of item 1 and .73 for the total scale in a Dutch sample of fibromyalgia patients (Hovens et al., 2009).

**Patient satisfaction, self-perceived intervention effects and the therapeutic relationship**

After the intervention, patient satisfaction and experience with the intervention, and self-perceived intervention effects were evaluated in a self-report questionnaire. At baseline and post-intervention, the expected/perceived patient-provider interaction was measured with the Internet-specific Therapeutic Relationship Questionnaire (ITRQ; Ferwerda et al., 2016). The ITRQ consists of 10 items rated on a 10-point Likert scale ranging from “totally disagree” to “completely agree”. Higher scores indicate a stronger therapeutic relationship. The ITRQ showed good reliability for the total scale (α = .89) and the two separate subscales; Internet-specific Time and Attention (Cronbach’s α = .92), and Internet-specific Reflection and Comfort (α = .87; Ferwerda et al., 2016). Additional instruments were applied to research cost-effectiveness (specified on clinicaltrials.gov) but will be reported elsewhere.

**Descriptive characteristics**

*Hand osteoarthritis*

A baseline self-report questionnaire was used to assess sociodemographic variables (age, sex [male/female], marital and relationship status, education) and disease- and treatment-related variables (duration of osteoarthritis, osteoarthritis in other joint sites, previously received treatments for osteoarthritis, physical and psychological comorbidities, and use of medication for these comorbidities [yes/no]). Next, data were used from a standard physical examination of painful hand, knee and hip joints on palpitation.

*Fibromyalgia*

A baseline self-report questionnaire was used to assess sociodemographic variables (age, sex [male/female], marital and relationship status, education) and disease- and treatment-related variables (duration of fibromyalgia, previously received treatments for fibromyalgia, physical and psychological comorbidities, and use of medication for these comorbidities [yes/no]). At the final assessment, patients were asked whether they had received any psychological treatment during the study period and, if so, for what reasons. Patients were also asked whether they had used medication for the psychological symptoms for which they received treatment.

*Both studies*

Internet use was assessed with 2 Likert-scale questions on internet use and internet use proficiency. Furthermore, personality traits (extraversion and neuroticism) were assessed with the 48-item Eysenck Personality Questionnaire short-form (EPQ-RSS; Sanderman et al., 2012). Items are rated as “yes/no”, where higher frequencies of “yes” indicate a stronger presence of the trait. The measure shows acceptable to good internal consistency for the Extraversion (α ≥ .72) and Neuroticism (α ≥ .81) scales across different populations (Sanderman et al., 2012).

**Definitions adverse events and serious adverse events**

Adverse events could be reported throughout the studies. An adverse event was defined as any undesirable experience occurring to a patient during the study, whether or not related to the intervention (International Council for Harmonisation of Technical Requirements for Pharmaceuticals for Human Use, 2025; Rozental et al., 2014). A serious adverse event was considered a medical occurrence that was either life-threatening, resulted in death, required initial or prolonged existing hospitalization (excluding elective hospital admissions), resulted in significant disability or incapacity, was a congenital anomaly or birth defect, or that was any other important medical event that could have led to the aforementioned outcomes had medical or surgical intervention not taken place (International Council for Harmonisation of Technical Requirements for Pharmaceuticals for Human Use, 2025; Rozental et al., 2014).

**Supplementary Appendix D. Baseline characteristics in the hand osteoarthritis study and fibromyalgia study**

**Table S2**

Baseline characteristics in the hand osteoarthritis study

|  | iCBT group  (*n* = 35) | CAU group  (*n* = 35) |
| --- | --- | --- |
| **Sociodemographic variables** |  |  |
| Age, in years, *mean* (*SD*) | 61.80 (7.39) | 62.97 (7.82) |
| Female sex, *n* (%) | 28 (80.0) | 27 (77.1) |
| Married / living together, *n* (%) | 29 (82.9) | 27 (77.1) |
| Not married / not living together,  but in a relationship, *n* (%) | 4 (11.4) | 3 (8.6) |
| Lower education^a^, *n* (%) | 16 (45.7) | 21 (60.0) |
|  |  |  |
| **Disease- / treatment-related variables** |  |  |
| Duration of hand osteoarthritis symptoms in years, *median* (IQR) | 4.00 (8.00) | 5.50 (8.50)^b^ |
| Years since diagnosis, *median* (IQR) | 0.00 (5.00) | 1.00 (6.00) |
| Exercise therapy / physical therapy: |  |  |
| Yes currently, *n* (%) | 4 (11.4) | 4 (11.4) |
| In the past, *n* (%) | 4 (11.4) | 8 (22.9) |
| Received injection in base of thumb, fingers, or wrist, *n* (%) | 2 (5.7) | 6 (17.1) |
| Underwent surgery on one or both hands, *n* (%) | 7 (20.0) | 12 (34.3) |
| Other diagnosed rheumatic diseases, *n* (%) | 13 (37.1) | 15 (42.9) |
| Physical comorbidities (excluding other rheumatic diseases), *n* (%) | 19 (54.3) | 18 (51.4) |
| Use of medication for physical comorbidities, *n* (%) | 19 (54.3) | 15 (42.9) |
| Psychological comorbidities, *n* (%) | 2 (5.7) | 2 (5.7) |
| Use of medication for psychological comorbidities, *n* (%) | 1 (2.9) | 0 (0.0) |
|  |  |  |
| **Physical examination** |  |  |
| Doyle Index: |  |  |
| Total score, *median* (IQR) | 10.50 (10.13)^c^ | 7.00 (13.00)^d^ |
| Hand joints, *median* (IQR) | 6.00 (6.00)^e^ | 4.00 (6.00) |
| Knee/hip joints^f^, *median* (IQR) | 0.00 (1.00)^c^ | 0.00 (0.00)^d^ |
| Grip strength left hand, in kg, *mean* (*SD*) | 22.26 (10.59)^e^ | 21.54 (9.52)^b^ |
| Grip strength right hand, in kg, *mean* (*SD*) | 23.26 (10.64)^e^ | 22.69 (10.67)^b^ |
| Pinch strength left hand, in kg, *mean* (*SD*) | 2.75 (1.58)^e^ | 2.73 (1.64)^b^ |
| Pinch strength right hand, in kg, *mean* (*SD*) | 2.79 (1.76)^g^ | 2.78 (1.67)^b^ |
| Diagnosis of hand osteoarthritis, *n* (%) | 32 (97)^h^ | 32 (91.4)^i^ |
|  |  |  |
| **Other characteristics** |  |  |
| Frequency of internet use: |  |  |
| (Almost) every day, *n* (%) | 26 (74.3) | 31 (88.6) |
| Multiple times per week, *n* (%) | 5 (14.3) | 4 (11.4) |
| Once per week, *n* (%) | 1 (2.9) | 0 (0.0) |
| Less than once per week, *n* (%) | 2 (5.7) | 0 (0.0) |
| (Almost) never, *n* (%) | 1 (2.9) | 0 (0.0) |
| Proficiency in internet use: |  |  |
| Very good, *n* (%) | 8 (22.9) | 8 (22.9) |
| Good, *n* (%) | 19 (54.3) | 17 (48.6) |
| Reasonable, *n* (%) | 8 (22.9) | 10 (28.6) |
| Bad, *n* (%) | 0 (0.0) | 0 (0.0) |
| EPQ-RSS: |  |  |
| Extraversion, *median* (IQR) | 8.0 (5.00) | 9.0 (4.00)^b^ |
| Neuroticism, *median* (IQR) | 3.0 (5.00) | 3.5 (5.00)^b^ |

**Note:** The Doyle Index is an articular index for the assessment of joint pain in osteoarthritis (Doyle et al., 1981). Higher scores indicate more severe pain. Ranges of total pain score: 0-144; ranges of pain score hand joints: 0-72; ranges of pain score knee/hip joints: 0-12. iCBT = internet-based cognitive-behavioral therapy; CAU = care-as-usual; *n* = number; IQR = interquartile range; kg = kilogram; *SD* = standard deviation; EPQ-RSS = Eysenck Personality Questionnaire short-form.

^a^Lower education included primary education to vocational education; the remaining participants received higher education, which included advanced secondary and tertiary education.

^b^*n* = 34.

^c^*n* = 28.

^d^*n* = 27.

^e^*n* = 33.

^f^Method of testing for knee pain was movement instead of pressure.

^g^*n* = 32. One outlier which was likely to be an error has been removed.

^h^ACR criteria for hand osteoarthritis were not met for one participant based on the physical examination, however, the diagnosis of radiographic signs of hand osteoarthritis was made by the treating rheumatologist; two participants did not complete the physical examination but did meet the ACR criteria for hand osteoarthritis based on the patients’ medical files.

^i^Three participants did not meet the ACR criteria based on the physical examination: one participant did meet the ACR criteria for hand osteoarthritis based on the patient medical file, and two received the diagnosis of radiographic signs of hand osteoarthritis from the treating rheumatologist.

**Table S3**

Baseline characteristics in the fibromyalgia study

|  | iCBT group  (*n* = 34) | Waitlist group  (*n* = 36) |
| --- | --- | --- |
| **Sociodemographic variables** |  |  |
| Age, in years, *mean* (*SD*) | 45.88 (11.97) | 46.94 (11.79) |
| Female sex, *n* (%) | 31 (91.2) | 33 (91.7) |
| Married / living together, *n* (%) | 22 (64.7) | 24 (66.7) |
| Not married / not living together,  but in a relationship, *n* (%) | 4 (11.8) | 6 (16.7) |
| Lower education^a^, *n* (%) | 23 (67.6) | 17 (47.2) |
|  |  |  |
| **Disease- / treatment-related variables** |  |  |
| Duration of fibromyalgia symptoms in years, *median* (IQR) | 12.50 (12.50) | 12.50 (13.00) |
| Years since diagnosis, *median* (IQR) | 5.00 (9.00) | 3.50 (7.75) |
| Exercise therapy / physical therapy: |  |  |
| Yes currently, *n* (%) | 8 (23.5)^b^ | 7 (19.4) |
| In the past, *n* (%) | 17 (50.0)^b^ | 22 (61.1) |
| Psychological treatment for fibromyalgia in the past, *n* (%) | 14 (41.2)^b^ | 15 (41.7) |
| Other diagnosed rheumatic diseases, *n* (%) | 8 (23.5)^b^ | 11 (30.6) |
| Physical comorbidities (excluding other rheumatic diseases), *n* (%) | 12 (35.2)^b^ | 17 (47.2) |
| Use of medication for physical comorbidities, *n* (%) | 10 (29.4)^b^ | 13 (36.1) |
| Psychological comorbidities, *n* (%) | 2 (5.9)^b^ | 7 (19.4) |
| Use of medication for psychological comorbidities, *n* (%) | 1 (2.9)^b^ | 4 (11.1) |
|  |  |  |
| **Other characteristics** |  |  |
| Frequency of internet use: |  |  |
| (Almost) every day, *n* (%) | 31 (91.2)^b^ | 35 (97.2) |
| Multiple times per week, *n* (%) | 2 (5.9)^b^ | 1 (2.8) |
| Once per week, *n* (%) | 0 (0)^b^ | 0 (0.0) |
| Less than once per week, *n* (%) | 0 (0)^b^ | 0 (0.0) |
| (Almost) never, *n* (%) | 0 (0)^b^ | 0 (0.0) |
| Proficiency in internet use: |  |  |
| Very good, *n* (%) | 21 (61.8)^b^ | 22 (61.1) |
| Good, *n* (%) | 8 (23.5)^b^ | 13 (36.1) |
| Reasonable, *n* (%) | 4 (11.8)^b^ | 1 (2.8) |
| Bad, *n* (%) | 0 (0.0)^b^ | 0 (0.0) |
| EPQ-RSS: |  |  |
| Extraversion, *median* (IQR) | 7.0 (7.00)^b^ | 7.0 (5.75) |
| Neuroticism, *median* (IQR) | 8.0 (5.00)^b^ | 8.0 (4.75) |

**Note:** iCBT = internet-based cognitive-behavioral therapy; *n* = number; *SD* = standard deviation; EPQ-RSS = Eysenck Personality Questionnaire short-form; IQR = interquartile range.

^a^Lower education included primary education to vocational education; the remaining participants received higher education, which included advanced secondary and tertiary education.

^b^*n* = 33.

**Supplementary Appendix E. Assumptions, means, difference in change scores, and effect sizes in the hand osteoarthritis and fibromyalgia studies**

**Assumptions**

*Hand osteoarthritis*

Pain coping scores were approximately normally distributed, with moderate skewness (Z = -2.72) and high kurtosis (Z = 7.12). The assumption of normality was not met for Physical Functioning (RAND-36), Role Limitations due to Personal or Emotional Problems (RAND-36), Social Functioning (RAND-36), Life Control (MPI-DLV), Support (MPI-DLV), Timeline Acute/Chronic (IPQ-R), and Consequences (IPQ-R). For these variables, a transformation was applied. A transformation did not improve the distribution of Physical Functioning (RAND-36), therefore the results without transformation were reported. No other assumptions were violated.

*Fibromyalgia*

Pain coping scores were approximately normally distributed, with moderate skewness (Z = -2.34) and low kurtosis (Z = 0.88). The assumption of normality was not met for Health Perceptions (RAND-36), Timeline Acute/Chronic (IPQ-R), and Transformation (PCI). For these variables, a transformation was applied. No other assumptions were violated.

**Table S4**

**Means, difference in change scores, and effect sizes (Cohen’s *d*) of primary and secondary outcome variables for each assessment in the hand osteoarthritis study**

| Outcome | Baseline | | Post-intervention | | | | 6-week follow-up | | | | 3-month follow-up | | | |
| --- | --- | --- | --- | --- | --- | --- | --- | --- | --- | --- | --- | --- | --- | --- |
|  | iCBT  *M* (*SD*)  *n* = 35 | CAU  *M* (*SD*)  *n* = 35 | iCBT  *M* (*SD*)  *n* = 24 | CAU  *M* (*SD*)  *n* = 35 | Diff. in change | *d* (95%CI) | iCBT  *M* (*SD*)  *n* = 18 | CAU  *M* (*SD*)  *n* = 35 | Diff. in change | *d* (95%CI) | iCBT  *M* (*SD*)  *n* = 17 | CAU  *M* (*SD*)  *n* = 34 | Diff. in change | *d* (95%CI) |
| **Primary outcome** |  |  |  |  |  |  |  |  |  |  |  |  |  |  |
| Pain Coping (VAS) | 6.9 (2.0) | 6.5 (1.8) | 7.3 (1.8) | 6.4 (2.0) | 0.29 | 0.14 (-0.38, 0.66) | 7.6 (1.7) | 6.2 (2.2) | 0.92 | 0.41 (-0.16, 0.99) | 6.8 (2.1) | 6.2 (2.1) | 0.26 | 0.12 (-0.47, 0.70) |
| **Secondary outcomes** |  |  |  |  |  |  |  |  |  |  |  |  |  |  |
| **Psychological functioning** |  |  |  |  |  |  |  |  |  |  |  |  |  |  |
| ***Well-being*** |  |  |  |  |  |  |  |  |  |  |  |  |  |  |
| Well-being (VAS) | 6.3 (1.9) | 6.3 (1.9) | 6.3 (1.8) | 6.0 (1.9) | 0.20 | 0.09 (-0.43, 0.61) | 6.8 (1.5) | 6.0 (2.3) | 1.01 | 0.46 (-0.11, 1.04) | 5.7 (1.7) | 6.1 (2.0) | -0.06 | -0.03 (-0.61, 0.56) |
| RAND-36 |  |  |  |  |  |  |  |  |  |  |  |  |  |  |
| Mental Composite Score | 71.4 (17.5) | 73.0 (15.4) | 72.0 (16.4) | 67.6 (21.1) | 6.45 | 0.42 (-0.11, 0.94) | 70.2 (17.4) | 69.4 (19.4) | 3.99 | 0.28 (-0.29, 0.85) | 70.8 (17.5) | 67.4 (19.4) | 8.08 | 0.43 (-0.16, 1.02) |
| Emotional Well-being | 74.4 (14.1) | 75.1 (13.6) | 76.2 (13.3)^a^ | 71.9 (15.7) | 5.81 | 0.46 (-0.07, 0.99) | 72.2 (12.8) | 73.6 (15.8) | 0.37 | 0.03 (-0.54, 0.60) | 75.3 (11.1) | 71.1 (15.7) | 7.41 | 0.58 (-0.02, 1.17) |
| MPI-DLV |  |  |  |  |  |  |  |  |  |  |  |  |  |  |
| Affective Distress | 2.0 (1.4) | 1.7 (1.1) | 1.8 (1.1)^a^ | 1.8 (1.1) | -0.35 | 0.26 (-0.27, 0.79) | 2.4 (1.3) | 1.7 (1.2) | 0.26 | -0.23 (-0.79, 0.35) | 1.8 (1.1) | 2.1 (1.2) | -0.85 | 0.81 (0.20, 1.41) |
| ***Coping and cognitions*** |  |  |  |  |  |  |  |  |  |  |  |  |  |  |
| ICQ |  |  |  |  |  |  |  |  |  |  |  |  |  |  |
| Helplessness | 10.7 (4.0) | 11.3 (3.5) | 9.8 (2.9)^a^ | 10.7 (3.3) | -0.14 | 0.05 (-0.47, 0.58) | 9.9 (2.8) | 11.9 (3.9) | -0.88 | 0.39 (-0.19, 0.96) | 10.8 (3.3) | 11.1 (3.7) | 0.79 | -0.32 (-0.90, 0.27) |
| Acceptance | 15.3 (3.8) | 15.3 (3.3) | 17.0 (3.6)^a^ | 15.8 (3.7) | 1.11 | 0.34 (-0.20, 0.86) | 16.3 (2.8) | 15.7 (3.4) | 1.10 | 0.32 (-0.25, 0.89) | 16.5 (3.7) | 15.3 (3.6) | 1.68 | 0.54 (-0.06, 1.13) |
| PCI |  |  |  |  |  |  |  |  |  |  |  |  |  |  |
| Active Pain Coping | 25.3 (6.0) | 25.5 (5.0) | 26.9 (5.7)^a^ | 26.9 (5.1) | 0.23 | 0.05 (-0.48, 0.58) | 27.2 (7.0) | 25.9 (5.1) | 1.18 | 0.23 (-0.34, 0.80) | 25.7 (5.2) | 26.1 (5.4) | 0.01 | 0.00 (-0.58, 0.59) |
| Transformation | 8.5 (3.0) | 8.9 (2.4) | 8.4 (2.8)^a^ | 9.2 (2.6) | -0.64 | -0.29 (-0.82, 0.24) | 9.2 (3.1) | 8.9 (2.8) | -0.19 | -0.08 (-0.65, 0.49) | 8.3 (2.9) | 8.8 (2.6) | -0.67 | -0.28 (-0.87, 0.31) |
| Distraction | 10.4 (3.6) | 10.5 (3.3) | 11.8 (3.5)^a^ | 11.3 (3.2) | 0.61 | 0.19 (-0.34, 0.71) | 11.5 (4.0) | 10.6 (3.1) | 1.01 | 0.28 (-0.29, 0.85) | 11.0 (2.7) | 10.8 (2.9) | 0.44 | 0.14 (-0.44, 0.73) |
| Reducing Demands | 6.4 (2.0) | 6.1 (2.0) | 6.6 (2.0)^a^ | 6.4 (1.8) | 0.27 | 0.12 (-0.41, 0.65) | 6.4 (1.9) | 6.3 (1.8) | 0.35 | 0.19 (-0.38, 0.76) | 6.4 (1.7) | 6.6 (1.7) | 0.24 | 0.12 (-0.47, 0.70) |
| Passive Pain Coping | 37.1 (9.5) | 34.7 (10.1) | 36.6 (6.3)^a^ | 36.8 (11.0) | -2.36 | 0.39 (-0.15, 0.92) | 36.1 (8.3) | 35.8 (10.3) | -2.04 | 0.33 (-0.24, 0.90) | 36.1 (7.8) | 37.5 (11.3) | -4.08 | 0.65 (0.05, 1.25) |
| Retreating | 12.6 (3.3) | 11.1 (4.1) | 12.7 (3.0)^a^ | 12.2 (4.4) | -1.09 | 0.48 (-0.06, 1.01) | 11.5 (3.0) | 11.5 (4.5) | -1.43 | 0.53 (-0.05, 1.11) | 11.8 (3.5) | 12.4 (4.9) | -2.08 | 0.82 (0.22, 1.43) |
| Worrying | 14.5 (5.7) | 14.5 (6.0) | 14.0 (3.8)^a^ | 15.3 (5.9) | -1.37 | 0.30 (-0.24, 0.82) | 14.7 (5.8) | 15.1 (5.7) | -0.77 | 0.15 (-0.42, 0.72) | 14.2 (4.9) | 15.4 (5.6) | -1.59 | 0.38 (-0.21, 0.96) |
| Resting | 10.0 (3.0) | 9.0 (2.6) | 9.9 (2.4)^a^ | 9.3 (2.5) | 0.09 | -0.05 (-0.57, 0.48) | 9.9 (3.0) | 9.2 (2.4) | 0.16 | -0.08 (-0.65, 0.49) | 10.1 (2.3) | 9.8 (2.7) | -0.41 | 0.21 (-0.38, 0.79) |
| IPQ-R |  |  |  |  |  |  |  |  |  |  |  |  |  |  |
| Identity | 4.6 (1.4) | 4.8 (1.5) | 4.3 (1.5)^a^ | 5.3 (1.8) | -0.72 | 0.50 (-0.03, 1.03) | 4.4 (1.5) | 5.3 (1.9) | -0.74 | 0.48 (-0.10, 1.05) | 4.6 (1.5) | 5.3 (1.8) | -0.59 | 0.34 (-0.25, 0.93) |
| Timeline Acute/Chronic | 27.5 (2.8) | 28.1 (2.3) | 28.0 (2.5)^a^ | 27.5 (3.0) | 0.86 | -0.29 (-0.82, 0.24) | 26.8 (3.8) | 28.4 (2.1) | -1.18 | 0.51 (-0.07, 1.08) | 28.4 (2.4) | 28.2 (2.2) | 0.79 | -0.35 (-0.94, 0.23) |
| Timeline Cyclical | 13.3 (2.8) | 13.4 (3.0) | 13.3 (2.2)^b^ | 13.7 (3.3) | -0.19 | 0.06 (-0.47, 0.59) | 13.4 (4.1) | 14.1 (2.5) | -0.63 | 0.20 (-0.37, 0.77) | 13.4 (3.1) | 13.9 (2.4) | -0.26 | 0.09 (-0.49, 0.68) |
| Consequences | 16.5 (4.3) | 17.2 (4.6) | 16.0 (4.5)^a^ | 17.0 (5.0) | -0.26 | 0.08 (-0.45, 0.61) | 15.5 (5.0) | 17.1 (5.0) | -1.55 | 0.52 (-0.06, 1.09) | 16.1 (4.2) | 17.3 (4.9) | -1.26 | 0.33 (-0.26, 0.92) |
| Personal Control | 18.7 (3.0) | 18.1 (3.1) | 19.0 (4.1)^a^ | 18.7 (3.1) | -0.27 | -0.08 (-0.61, 0.45) | 17.9 (3.7) | 17.3 (4.0) | 0.10 | 0.03 (-0.54, 0.60) | 16.8 (4.2) | 17.1 (4.0) | -0.62 | -0.16 (-0.74, 0.43) |
| Outcome | Baseline | | Post-intervention | | | | 6-week follow-up | | | | 3-month follow-up | | | |
|  | iCBT  *M* (*SD*)  *n* = 35 | CAU  *M* (*SD*)  *n* = 35 | iCBT  *M* (*SD*)  *n* = 24 | CAU  *M* (*SD*)  *n* = 35 | Diff. in change | *d* (95%CI) | iCBT  *M* (*SD*)  *n* = 18 | CAU  *M* (*SD*)  *n* = 35 | Diff. in change | *d* (95%CI) | iCBT  *M* (*SD*)  *n* = 17 | CAU  *M* (*SD*)  *n* = 34 | Diff. in change | *d* (95%CI) |
| Treatment Control | 13.8 (2.8) | 14.1 (2.6) | 13.7 (3.2)^b^ | 13.1 (2.5) | 0.59 | 0.24 (-0.30, 0.77) | 13.0 (3.3) | 12.5 (2.7) | 0.60 | 0.22 (-0.35, 0.79) | 12.8 (3.1) | 12.5 (2.4) | 0.59 | 0.24 (-0.34, 0.83) |
| Emotional Rep | 15.1 (4.4) | 15.1 (4.4) | 14.1 (5.1)^b^ | 14.5 (4.9) | -0.77 | 0.18 (-0.35, 0.72) | 13.5 (5.3) | 14.7 (4.9) | -2.15 | 0.52 (-0.06, 1.09) | 13.3 (4.4) | 15.3 (5.2) | -3.41 | 0.76 (0.15, 1.36) |
| Illness Coherence | 19.1 (3.6) | 18.6 (4.2) | 20.2 (3.6)^b^ | 19.6 (3.2) | -0.50 | -0.14 (-0.67, 0.40) | 19.6 (4.3) | 19.3 (4.2) | -0.69 | -0.19 (-0.76, 0.38) | 19.7 (4.2) | 19.2 (3.3) | -0.06 | -0.02 (-0.60, 0.57) |
| MPI-DLV |  |  |  |  |  |  |  |  |  |  |  |  |  |  |
| Life Control | 4.4 (1.2) | 4.5 (1.2) | 4.4 (1.0)^a^ | 4.3 (1.2) | 0.21 | 0.17 (-0.36, 0.70) | 4.0 (1.2) | 4.4 (1.0) | -0.18 | -0.18 (-0.74, 0.40) | 4.7 (0.9) | 4.0 (1.0) | 1.00 | 0.98 (0.36, 1.58) |
| **Physical functioning** |  |  |  |  |  |  |  |  |  |  |  |  |  |  |
| Pain (VAS) | 5.1 (2.4) | 5.2 (2.2) | 4.5 (2.4) | 5.1 (2.2) | -0.66 | 0.28 (-0.25, 0.80) | 4.2 (2.4) | 4.8 (2.2) | -0.85 | 0.45 (-0.13, 1.03) | 4.8 (2.6) | 5.3 (2.2) | -0.68 | 0.30 (-0.29, 0.88) |
| AUSCAN |  |  |  |  |  |  |  |  |  |  |  |  |  |  |
| Pain | 9.4 (4.1) | 9.2 (3.9) | 9.8 (4.4)^a^ | 9.6 (3.8) | -0.36 | 0.13 (-0.40, 0.65) | 9.4 (3.8) | 9.5 (3.5) | -0.54 | 0.24 (-0.34, 0.81) | 10.0 (4.2) | 10.2 (3.7) | -0.53 | 0.15 (-0.43, 0.74) |
| Disability | 17.4 (9.1) | 17.9 (7.1) | 17.9 (8.7)^a^ | 18.6 (7.5) | -0.35 | 0.07 (-0.45, 0.60) | 17.7 (9.0) | 17.8 (7.1) | -0.66 | 0.15 (-0.42, 0.72) | 17.8 (8.3) | 19.8 (7.0) | -2.90 | 0.65 (0.05, 1.24) |
| MPI-DLV |  |  |  |  |  |  |  |  |  |  |  |  |  |  |
| Pain Severity | 2.7 (1.2) | 2.9 (1.3) | 3.1 (1.4)^a^ | 2.8 (1.4) | 0.57 | -0.46 (-0.99, 0.07) | 2.8 (1.2) | 2.8 (1.5) | 0.27 | -0.23 (-0.80, 0.34) | 2.6 (1.3) | 3.1 (1.3) | -0.24 | 0.18 (-0.41, 0.76) |
| RAND-36 |  |  |  |  |  |  |  |  |  |  |  |  |  |  |
| Physical Composite Score | 58.8 (20.2) | 52.7 (19.4) | 58.5 (17.7) | 54.0 (21.2) | 0.01 | 0.00 (-0.52, 0.52) | 59.1 (22.3) | 56.7 (21.3) | -2.57 | -0.21 (-0.78, 0.36) | 60.7 (16.4) | 52.3 (20.4) | 2.46 | 0.24 (-0.35, 0.82) |
| Physical Functioning | 68.9 (17.9) | 63.3 (23.9) | 67.1 (17.7) | 64.0 (24.0) | -1.34 | -0.08 (-0.60, 0.44) | 68.9 (19.4) | 65.7 (22.1) | -2.15 | -0.17 (-0.74, 0.40) | 66.8 (19.3) | 60.0 (21.7) | 1.03 | 0.07 (-0.51, 0.65) |
| Bodily Pain | 58.1 (17.5) | 55.9 (18.1) | 63.9 (12.4) | 56.4 (16.5) | 4.92 | 0.32 (-0.20, 0.84) | 61.8 (16.3) | 58.8 (18.3) | 0.34 | 0.03 (-0.54, 0.59) | 60.6 (16.4) | 56.5 (18.8) | 1.62 | 0.10 (-0.48, 0.68) |
| Energy/Fatigue | 55.0 (18.2) | 55.3 (15.1) | 55.7 (18.3)^a^ | 57.1 (17.6) | -0.12 | -0.01 (-0.53, 0.52) | 57.5 (20.4) | 56.3 (15.7) | 0.39 | 0.03 (-0.54, 0.60) | 55.3 (18.2) | 53.7 (15.3) | 0.88 | 0.06 (-0.53, 0.64) |
| Health Change | 44.3 (21.1) | 35.7 (16.4) | 55.2 (28.5) | 42.9 (24.7) | 4.32 | 0.16 (-0.36, 0.68) | 52.8 (22.5) | 44.3 (22.8) | 1.15 | 0.05 (-0.52, 0.62) | 50.0 (19.8) | 34.6 (17.4) | 11.76 | 0.57 (-0.03, 1.16) |
| **Impact on daily life** |  |  |  |  |  |  |  |  |  |  |  |  |  |  |
| MPI-DLV |  |  |  |  |  |  |  |  |  |  |  |  |  |  |
| Interference | 1.9 (1.4) | 1.9 (1.1) | 1.9 (1.1)^a^ | 2.0 (1.1) | 0.10 | -0.12 (-0.65, 0.41) | 1.8 (1.2) | 2.0 (1.3) | -0.06 | 0.06 (-0.51, 0.63) | 1.8 (1.2) | 2.1 (1.2) | -0.23 | 0.28 (-0.30, 0.87) |
| Support | 3.1 (1.6)^c^ | 3.5 (1.4)^d^ | 3.0 (1.4)^e^ | 3.3 (1.5)^f^ | 0.40 | 0.38 (-0.20, 0.95) | 2.9 (1.8)^g^ | 3.3 (1.7)^f^ | 0.24 | 0.20 (-0.41, 0.82) | 2.5 (1.6) | 3.5 (1.5)^h^ | -0.21 | -0.19 (-0.81, 0.43) |
| RAND-36 |  |  |  |  |  |  |  |  |  |  |  |  |  |  |
| Role Limitations due to Physical Health Problems | 51.4 (44.5) | 35.0 (39.4) | 51.0 (40.7) | 40.7 (40.3) | -2.59 | -0.08 (-0.60, 0.44) | 51.4 (42.4) | 48.6 (42.4) | -12.18 | -0.38 (-0.95, 0.20) | 60.3 (35.4) | 41.9 (40.2) | 1.47 | 0.05 (-0.53, 0.64) |
| General Health Perceptions | 56.9 (19.2) | 56.7 (16.7) | 52.6 (15.7)^a^ | 54.7 (19.3) | 0.04 | 0.00 (-0.52, 0.53) | 54.2 (21.2) | 53.6 (16.3) | 3.70 | 0.33 (-0.24, 0.91) | 55.0 (18.4) | 50.7 (17.0) | 5.74 | 0.42 (-0.17, 1.01) |
| Role Limitations due to Personal or Emotional Problems | 80.0 (33.5) | 81.0 (35.5) | 75.0 (39.6) | 67.6 (42.4) | 9.17 | 0.22 (-0.30, 0.74) | 70.4 (36.0) | 70.5 (41.8) | 6.77 | 0.20 (-0.38, 0.76) | 74.5 (41.7) | 68.6 (41.8) | 16.67 | 0.36 (-0.23, 0.94) |
| Social Functioning | 76.1 (20.9) | 80.7 (19.5) | 80.7 (16.9) | 73.6 (23.0) | 10.79 | 0.50 (-0.03, 1.02) | 80.6 (17.8) | 77.1 (20.0) | 8.43 | 0.38 (-0.19, 0.95) | 77.9 (17.4) | 76.1 (20.7) | 7.35 | 0.32 (-0.27, 0.91) |

**Note:** A positive effect size indicates a change since baseline in favor of the intervention group and a negative effect size indicates a change since baseline in favor of the control group. Due to non-normal distributions, several transformed variables were used in the linear mixed model analyses; results of the untransformed variables are displayed in this table. iCBT = internet-based cognitive-behavioral therapy; CAU = care-as-usual; diff. in change = difference in change (compared to baseline) between the intervention and control group; *M* = mean; *n* = number; *SD* = standard deviation; CI = confidence interval; VAS = visual analogue scale; RAND-36 = RAND 36-item Health Survey; MPI-DLV = Multidimensional Pain Inventory-Dutch Language Version; ICQ = Illness Cognition Questionnaire; PCI = Pain Coping Inventory; IPQ-R = Illness Perception Questionnaire-Revised; AUSCAN = Australian/Canadian Hand Osteoarthritis Index.

^a^*n* = 23.

^b^*n* = 22.

^c^*n* = 32.

^d^*n* = 30.

^e^*n* = 21.

^f^*n* = 29.

^g^*n* = 16.

^h^*n* = 28.

**Table S5**

**Means, difference in change scores, and effect sizes (Cohen’s *d*) of primary and secondary outcome variables for each assessment in the fibromyalgia study**

| Outcome | Baseline | | Post-intervention | | | | 6-week follow-up | | | | 3-month follow-up | | | |
| --- | --- | --- | --- | --- | --- | --- | --- | --- | --- | --- | --- | --- | --- | --- |
|  | iCBT  *M* (*SD*)  *n* = 33 | Waitlist  *M* (*SD*)  *n* = 36 | iCBT  *M* (*SD*)  *n* = 29 | Waitlist  *M* (*SD*)  *n* = 35 | Diff. in change | *d* (95%CI) | iCBT  *M* (*SD*)  *n* = 24 | Waitlist  *M* (*SD*)  *n* = 34 | Diff. in change | *d* (95%CI) | iCBT  *M* (*SD*)  *n* = 22 | Waitlist  *M* (*SD*)  *n* = 34 | Diff. in change | *d* (95%CI) |
| **Primary outcome** |  |  |  |  |  |  |  |  |  |  |  |  |  |  |
| Pain Coping (VAS) | 5.5 (1.8) | 5.3 (1.7) | 6.7 (1.5) | 5.3 (1.7) | 1.25 | 0.60 (0.10, 1.11) | 6.5 (2.2) | 6.3 (1.9) | 0.27 | 0.12 (-0.40, 0.64) | 6.2 (2.4) | 5.5 (2.1) | 0.85 | 0.30 (-0.24, 0.84) |
| **Secondary outcomes** |  |  |  |  |  |  |  |  |  |  |  |  |  |  |
| **Psychological functioning** |  |  |  |  |  |  |  |  |  |  |  |  |  |  |
| ***Well-being*** |  |  |  |  |  |  |  |  |  |  |  |  |  |  |
| Well-being (VAS) | 5.3 (1.5) | 5.1 (1.9) | 6.2 (1.4) | 5.2 (1.4) | 0.77 | 0.34 (-0.16, 0.83) | 6.4 (1.6) | 5.4 (1.8) | 0.68 | 0.31 (-0.22, 0.83) | 6.1 (2.0) | 5.1 (1.6) | 0.79 | 0.30 (-0.24, 0.84) |
| RAND-36 |  |  |  |  |  |  |  |  |  |  |  |  |  |  |
| Mental Composite Score | 49.9 (19.8) | 45.5 (20.5) | 57.0 (18.5)^a^ | 50.4 (18.7) | 0.22 | 0.01 (-0.49, 0.51) | 62.5 (17.8) | 53.1 (19.4) | 3.86 | 0.19 (-0.34, 0.71) | 58.3 (24.6) | 52.3 20.8) | 1.90 | 0.10 (-0.44, 0.64) |
| Emotional Well-being | 61.2 (17.7) | 56.6 (16.7) | 67.1 (14.1)^b^ | 63.3 (18.5) | -3.06 | -0.22 (-0.72, 0.28) | 68.0 (15.6) | 64.2 (18.3) | -0.22 | -0.01 (-0.54, 0.51) | 62.4 (20.7) | 64.0 (18.4) | -4.56 | -0.31 (-0.85, 0.23) |
| MPI-DLV |  |  |  |  |  |  |  |  |  |  |  |  |  |  |
| Affective Distress | 2.6 (1.3) | 3.0 (1.0) | 2.2 (1.1)^b^ | 2.7 (1.3) | -0.08 | 0.06 (-0.44, 0.56) | 2.2 (1.2) | 2.8 (1.3) | -0.26 | 0.20 (-0.33, 0.72) | 2.5 (1.6) | 3.0 (1.1) | -0.29 | 0.23 (-0.31, 0.77) |
| ***Coping and cognitions*** |  |  |  |  |  |  |  |  |  |  |  |  |  |  |
| ICQ |  |  |  |  |  |  |  |  |  |  |  |  |  |  |
| Helplessness | 15.5 (3.8) | 15.6 (3.3) | 13.9 (4.2)^b^ | 15.0 (3.2)^c^ | -0.69 | 0.26 (-0.25, 0.77) | 13.1 (3.3) | 14.3 (3.5) | -0.68 | 0.25 (-0.28, 0.77) | 13.3 (4.2) | 14.1 (3.4) | -0.66 | 0.23 (-0.31, 0.77) |
| Acceptance | 13.3 (3.9) | 12.7 (3.4) | 15.6 (4.7)^b^ | 13.6 (2.8)^c^ | 1.20 | 0.45 (-0.06, 0.96) | 15.0 (4.2) | 14.1 (3.1) | 0.26 | 0.10 (-0.42, 0.62) | 15.6 (5.0) | 13.7 (2.9) | 1.33 | 0.50 (-0.04, 1.05) |
| PCI |  |  |  |  |  |  |  |  |  |  |  |  |  |  |
| Active Pain Coping | 28.5 (5.5) | 28.4 (4.0) | 28.7 (4.4)^b^ | 27.3 (3.7)^c^ | 0.81 | 0.19 (-0.32, 0.70) | 28.7 (4.5) | 26.9 (3.8) | 1.35 | 0.33 (-0.20, 0.86) | 28.3 (4.6) | 26.7 (4.3) | 1.64 | 0.49 (-0.06, 1.03) |
| Transformation | 9.1 (3.2) | 9.3 (2.1) | 8.7 (2.7)^b^ | 8.8 (2.9)^c^ | -0.35 | -0.11 (-0.62, 0.39) | 9.4 (2.8) | 8.3 (2.3) | 1.22 | 0.49 (-0.04, 1.02) | 8.8 (2.3) | 8.7 (2.5) | 0.56 | 0.24 (-0.30, 0.78) |
| Distraction | 12.4 (2.8) | 12.5 (2.6) | 12.9 (2.7)^b^ | 12.2 (2.8)^c^ | 0.83 | 0.38 (-0.13, 0.89) | 12.5 (2.6) | 11.8 (2.3) | 0.87 | 0.37 (-0.16, 0.89) | 13.0 (2.9) | 11.9 (2.3) | 1.46 | 0.73 (0.18, 1.28) |
| Reducing Demands | 7.0 (2.0) | 6.5 (1.6) | 7.1 (1.1)^b^ | 6.3 (1.8)^c^ | 0.32 | 0.15 (-0.35, 0.66) | 6.8 (1.7) | 6.8 (1.5) | -0.74 | -0.34 (-0.87, 0.19) | 6.5 (1.3) | 6.1 (1.8) | -0.37 | -0.19 (-0.73, 0.35) |
| Passive Pain Coping | 46.1 (8.5) | 47.3 (9.9) | 43.2 (7.0)^b^ | 45.5 (10.0)^c^ | -0.97 | 0.16 (-0.35, 0.66) | 44.0 (8.1) | 44.9 (10.2) | 0.16 | -0.03 (-0.55, 0.50) | 44.5 (6.7) | 45.2 (9.9) | -0.22 | 0.03 (-0.50, 0.57) |
| Retreating | 14.9 (3.4) | 16.6 (4.2) | 15.0 (3.0)^b^ | 15.8 (4.3)^c^ | 1.13 | -0.51 (-1.02, 0.01) | 14.9 (3.8) | 15.3 (4.0) | 1.26 | -0.46 (-0.99, 0.07) | 16.5 (3.0) | 15.5 (4.2) | 2.40 | -0.95 (-1.52, -0.39) |
| Worrying | 19.3 (5.4) | 18.6 (5.7) | 16.7 (5.2)^b^ | 17.9 (6.0)^c^ | -2.47 | 0.53 (0.02, 1.05) | 17.4 (5.1) | 17.5 (5.7) | -1.38 | 0.40 (-0.13, 0.93) | 16.6 (4.6) | 17.6 (5.4) | -2.40 | 0.59 (0.04, 1.14) |
| Resting | 11.9 (3.1) | 12.2 (3.0) | 11.6 (2.7)^b^ | 11.9 (3.4)^c^ | 0.37 | -0.15 (-0.66, 0.35) | 11.7 (2.8) | 12.1 (2.9) | 0.28 | -0.12 (-0.64, 0.41) | 11.4 (2.4) | 12.1 (3.2) | -0.22 | 0.09 (-0.45, 0.62) |
| IPQ-R |  |  |  |  |  |  |  |  |  |  |  |  |  |  |
| Identity | 8.3 (2.3) | 8.5 (2.3) | 8.7 (1.9)^b^ | 8.2 (2.3)^c^ | 0.74 | -0.35 (-0.86, 0.16) | 8.0 (2.1) | 8.2 (2.3) | 0.11 | -0.05 (-0.58, 0.47) | 7.9 (2.4) | 8.6 (2.6) | -0.39 | 0.18 (-0.36, 0.72) |
| Timeline Acute/Chronic | 26.3 (3.8) | 26.1 (2.9) | 26.9 (3.2)^b^ | 26.4 (3.4)^c^ | -0.40 | 0.15 (-0.36, 0.65) | 26.4 (3.5) | 26.5 (3.2) | -1.17 | 0.42 (-0.12, 0.94) | 26.7 (3.7) | 26.2 (3.5) | -0.71 | 0.26 (-0.28, 0.80) |
| Timeline Cyclical | 15.2 (2.8) | 14.9 (2.8) | 14.7 (2.2)^b^ | 14.9 (3.1)^c^ | -0.62 | 0.20 (-0.30, 0.71) | 14.6 (2.1) | 14.6 (3.2) | -0.40 | 0.14 (-0.38, 0.66) | 15.0 (2.5) | 15.1 (2.8) | -0.40 | 0.15 (-0.39, 0.69) |
| Consequences | 20.2 (4.0) | 21.8 (3.7) | 20.3 (4.2)^b^ | 22.2 (3.6)^c^ | 0.03 | -0.01 (-0.52, 0.49) | 19.7 (3.9) | 22.1 (4.1) | -0.48 | 0.18 (-0.35, 0.70) | 19.5 (4.1) | 21.6 (4.1) | -0.45 | 0.16 (-0.38, 0.70) |
| Personal Control | 19.9 (3.5) | 19.9 (3.4) | 19.6 (4.4)^b^ | 19.5 (4.3)^c^ | -0.06 | -0.02 (-0.52, 0.49) | 19.0 (4.3) | 19.3 (3.9) | -0.80 | -0.23 (-0.75, 0.30) | 19.8 (4.6) | 19.3 (3.7) | 0.17 | 0.06 (-0.48, 0.59) |
| Outcome | Baseline | | Post-intervention | | | | 6-week follow-up | | | | 3-month follow-up | | | |
|  | iCBT  *M* (*SD*)  *n* = 33 | Waitlist  *M* (*SD*)  *n* = 36 | iCBT  *M* (*SD*)  *n* = 29 | Waitlist  *M* (*SD*)  *n* = 35 | Diff. in change | *d* (95%CI) | iCBT  *M* (*SD*)  *n* = 24 | Waitlist  *M* (*SD*)  *n* = 34 | Diff. in change | *d* (95%CI) | iCBT  *M* (*SD*)  *n* = 22 | Waitlist  *M* (*SD*)  *n* = 34 | Diff. in change | *d* (95%CI) |
| Treatment Control | 15.5 (2.6) | 14.8 (2.6) | 14.3 (2.9)^b^ | 14.3 (3.0)^c^ | -0.68 | -0.27 (-0.78, 0.24) | 13.8 (3.0) | 14.5 (3.0) | -1.56 | -0.63 (-1.16, -0.09) | 14.3 (2.9) | 14.3 (3.3) | -0.73 | -0.28 (-0.82, 0.26) |
| Emotional Rep | 17.2 (4.2) | 18.3 (5.0) | 16.0 (5.1)^b^ | 17.9 (4.4)^c^ | -0.43 | 0.10 (-0.41, 0.61) | 16.3 (5.4) | 17.8 (4.8) | -0.20 | 0.05 (-0.47, 0.58) | 15.8 (4.4) | 18.0 (4.6) | -1.09 | 0.27 (-0.28, 0.80) |
| Illness Coherence | 15.6 (4.4) | 15.7 (4.4) | 16.7 (4.7)^b^ | 15.6 (4.8)^c^ | 0.96 | 0.31 (-0.20, 0.82) | 17.3 (5.5) | 15.5 (4.7) | 1.05 | 0.34 (-0.19, 0.86) | 17.2 (4.2) | 15.7 (4.6) | 0.99 | 0.33 (-0.21, 0.87) |
| MPI-DLV |  |  |  |  |  |  |  |  |  |  |  |  |  |  |
| Life Control | 3.7 (1.0) | 3.3 (1.0) | 3.9 (0.9)^b^ | 3.5 (1.1)^c^ | 0.04 | 0.04 (-0.46, 0.55) | 3.9 (1.1) | 3.4 (1.0) | 0.14 | 0.13 (-0.40, 0.65) | 3.7 (1.1) | 3.2 (1.1) | 0.27 | 0.26 (-0.28, 0.80) |
| **Physical functioning** |  |  |  |  |  |  |  |  |  |  |  |  |  |  |
| Pain (VAS) | 6.9 (1.6) | 6.8 (1.5) | 6.0 (1.8) | 6.1 (1.6) | -0.18 | 0.10 (-0.39, 0.59) | 5.4 (1.9) | 5.7 (2.1) | -0.50 | 0.20 (-0.32, 0.73) | 5.0 (2.4) | 6.2 (1.6) | -1.35 | 0.63 (0.08, 1.18) |
| MPI-DLV |  |  |  |  |  |  |  |  |  |  |  |  |  |  |
| Pain Severity | 4.0 (1.0) | 4.0 (1.2) | 3.6 (1.3)^b^ | 3.7 (1.2) | -0.08 | 0.07 (-0.43, 0.57) | 3.3 (1.3) | 3.4 (1.3) | -0.09 | 0.06 (-0.46, 0.58) | 3.4 (1.3) | 3.7 (1.0) | -0.25 | 0.19 (-0.35, 0.73) |
| RAND-36 |  |  |  |  |  |  |  |  |  |  |  |  |  |  |
| Physical Composite Score | 34.9 (16.2) | 34.7 (15.9) | 45.6 (20.2)^a^ | 38.5 (15.8) | 5.39 | 0.42 (-0.09, 0.92) | 46.9 (17.9) | 41.5 (15.8) | 4.01 | 0.29 (-0.24, 0.82) | 52.8 (22.8) | 41.3 (13.9) | 9.72 | 0.61 (0.06, 1.16) |
| Physical Functioning | 44.1 (20.5) | 51.5 (19.4) | 53.6 (21.7)^a^ | 52.9 (20.5) | 4.93 | 0.33 (-0.18, 0.83) | 56.7 (15.9) | 56.2 (20.7) | 3.36 | 0.21 (-0.32, 0.73) | 58.0 (22.2) | 55.6 (20.5) | 5.57 | 0.35 (-0.19, 0.89) |
| Bodily Pain | 35.9 (17.6) | 39.7 (20.6) | 50.4 (17.1)^b^ | 46.7 (18.1) | 7.87 | 0.38 (-0.13, 0.89) | 48.9 (16.1) | 47.4 (19.4) | 4.45 | 0.22 (-0.31, 0.74) | 57.2 (20.4) | 46.9 (14.6) | 12.61 | 0.57 (0.02, 1.11) |
| Energy/Fatigue | 31.5 (14.7) | 31.7 (15.5) | 40.4 (20.8)^b^ | 34.3 (14.0) | 5.82 | 0.38 (-0.13, 0.89) | 42.5 (20.6) | 34.9 (15.0) | 7.73 | 0.59 (0.05, 1.12) | 41.6 (22.3) | 35.3 (15.3) | 7.73 | 0.49 (-0.06, 1.03) |
| Health Change | 33.3 (27.7) | 43.8 (25.6) | 55.2 (34.3) | 45.7 (30.6) | 18.40 | 0.53 (0.03, 1.03) | 56.3 (29.7) | 45.6 (30.4) | 17.59 | 0.55 (0.01, 1.07) | 53.4 (31.1) | 44.1 (26.2) | 19.72 | 0.66 (0.11, 1.21) |
| **Impact on daily life** |  |  |  |  |  |  |  |  |  |  |  |  |  |  |
| MPI-DLV |  |  |  |  |  |  |  |  |  |  |  |  |  |  |
| Interference | 3.6 (1.1) | 3.8 (1.2) | 3.2 (1.0)^b^ | 3.5 (1.1) | -0.04 | 0.05 (-0.46, 0.55) | 3.1 (1.0) | 3.6 (1.0) | -0.25 | 0.30 (-0.23, 0.82) | 3.0 (1.1) | 3.7 (1.1) | -0.63 | 0.63 (0.08, 1.17) |
| Support | 3.6 (1.7)^d^ | 3.2 (1.4)^e^ | 3.4 (1.5)^f^ | 3.4 (1.2)^a^ | -0.22 | -0.21 (-0.78, 0.36) | 3.2 (1.6)^g^ | 2.9 (1.2)^h^ | 0.33 | 0.30 (-0.30, 0.90) | 3.1 (1.5)^i^ | 3.0 (1.3)^b^ | -0.19 | -0.21 (-0.82, 0.40) |
| RAND-36 |  |  |  |  |  |  |  |  |  |  |  |  |  |  |
| Role Limitations due to Physical Health Problems | 24.2 (34.5) | 15.3 (24.8) | 34.8 (39.3)^a^ | 20.0 (27.7) | 4.82 | 0.17 (-0.33, 0.66) | 34.4 (36.0) | 26.5 (29.5) | 1.47 | 0.05 (-0.48, 0.57) | 48.9 (38.2) | 25.0 (32.0) | 16.58 | 0.46 (-0.08, 1.00) |
| General Health Perceptions | 35.5 (17.2) | 32.4 (17.5) | 43.1 (20.7)^b^ | 34.3 (19.5) | 3.72 | 0.25 (-0.25, 0.76) | 47.7 (21.9) | 35.9 (18.7) | 6.74 | 0.43 (-0.10, 0.96) | 47.0 (22.9) | 37.6 (14.0) | 4.10 | 0.23 (-0.31, 0.77) |
| Role Limitations due to Personal or Emotional Problems | 58.6 (44.9) | 45.4 (48.6) | 59.5 (42.9)^a^ | 52.4 (45.2) | -10.48 | -0.18 (-0.68, 0.32) | 70.8 (42.1) | 56.9 (41.5) | -3.27 | -0.06 (-0.58, 0.47) | 66.7 (42.4) | 52.9 (47.9) | -1.87 | -0.04 (-0.58, 0.50) |
| Social Functioning | 48.5 (26.7) | 48.3 (22.6) | 63.4 (17.0)^b^ | 51.4 (24.4) | 10.32 | 0.52 (0.01, 1.03) | 68.8 (16.1) | 56.6 (25.8) | 11.18 | 0.53 (-0.01, 1.06) | 62.5 (25.9) | 57.0 (23.7) | 6.32 | 0.27 (-0.28, 0.80) |
| FIQ | 61.2 (12.9) | 63.4 (15.4) | 53.8 (14.0) | 60.1 (13.4) | -2.89 | 0.25 (-0.25, 0.74) | 48.6 (15.3) | 56.7 (18.4) | -4.71 | 0.32 (-0.21, 0.84) | 48.5 (19.2) | 56.6 (13.8) | -5.02 | 0.37 (-0.17, 0.91) |

**Note:** A positive effect size indicates a change since baseline in favor of the treatment group and a negative effect size indicates a change since baseline in favor of the control group. Due to non-normal distributions, several transformed variables were used in the linear mixed model analyses; results of the untransformed variables are displayed in this table. iCBT = internet-based cognitive-behavioral therapy; diff. in change = difference in change (compared to baseline) between the treatment and control group; *M* = mean; *n* = number; *SD* = standard deviation; CI = confidence interval; VAS = visual analogue scale; RAND-36 = RAND 36-item Health Survey; MPI-DLV = Multidimensional Pain Inventory-Dutch Language Version; ICQ = Illness Cognition Questionnaire; PCI = Pain Coping Inventory; IPQ-R = Illness Perception Questionnaire-Revised; FIQ = Fibromyalgia Impact Questionnaire.

^a^n = 28.

^b^n = 27.

^c^n = 34.

^d^n = 25.

^e^n = 29.

^f^n = 21.

^g^n = 19.

^h^n = 26.

^i^n = 17.

**Supplementary Appendix F. Patient satisfaction in the hand osteoarthritis and fibromyalgia studies**

**Patient satisfaction**

At baseline, patients in both studies had overall rather positive expectations of the therapeutic relationship as reflected by ITRQ scale scores (Tables S6 and S7 in Supplementary Appendix F). At post-intervention, patients from the intervention groups in both studies rated the therapeutic relationship as generally rather positive, especially items concerning time lag aspects in communication and receiving sufficient attention by the therapist (ITRQ Time and Attention). User-friendliness was rated 6.23 (*SD* = 2.11) and 7.08 (*SD* = 1.70) out of 10 in the hand osteoarthritis study and fibromyalgia study, respectively. In both studies, intervention completers rated the intervention, user-friendliness and post-intervention ITRQ dimensions higher than non-completers (Supplementary Appendix F, Tables S6 and S7), although differences in ratings were not statistically tested.

When asked what could be improved in the online program, in both studies, some patients commented that they wished there was more personal (live) contact with the therapist (12.8% [6/47]), the intervention was more easily accessible via a smartphone (10.6% [5/47]), the website was more user-friendly (10.6% [5/47]), there would be less focus on psychological/emotional exercises (4.3% [2/47]), and the online program would be explained more clearly (4.3% [2/47]). In the fibromyalgia study, two users (7.7% [2/26]) suggested adding more support with physical exercise or physiotherapy exercise. In the hand osteoarthritis study and fibromyalgia study, 42.9% (9/21) and 57.7% (15/26) would recommend the intervention to a relative or friend with a chronic pain condition, while 42.9% (9/21) and 19.2% (5/26) answered neutrally to this item, respectively. Furthermore, 42.9% (9/21) and 50.0% (13/26) of patients believed the intervention would have positive long-term effects, while 47.6% (10/21) and 26.9% (7/26) answered neutrally to this item in the hand osteoarthritis study and fibromyalgia study, respectively.

**Table S6**

**Patient satisfaction for the iCBT group, and for completers and non-completers separately in the hand osteoarthritis study**

|  | iCBT | |  | Completers | |  | Non-completers | |
| --- | --- | --- | --- | --- | --- | --- | --- | --- |
|  | *M* (*SD*; range) | *n* |  | *M* (*SD*; range) | *n* |  | *M* (*SD*; range) | *n* |
| ***Baseline*** |  |  |  |  |  |  |  |  |
| ITRQ Time and Attention | 6.94 (1.42; 3.75-9.50) | 35 |  | 7.16 (1.31; 5.00-9.25) | 17 |  | 6.72 (1.52; 3.75-9.50) | 18 |
| ITRQ Reflection and Comfort | 6.30 (1.46; 2.75-10.00) | 35 |  | 6.00 (1.63; 2.75-8.75) | 17 |  | 6.58 (1.26; 4.25-10.00) | 18 |
| ITRQ Total Scale | 6.65 (1.12; 4.22-8.89) | 35 |  | 6.63 (1.14; 4.67-8.89) | 17 |  | 6.67 (1.12; 4.22-8.56) | 18 |
| ***Post-intervention*** |  |  |  |  |  |  |  |  |
| ITRQ Time and Attention | 7.19 (1.83; 3.25-10.00) | 22 |  | 7.50 (1.74; 4.00-10.00) | 17 |  | 6.15 (1.93; 3.25-8.00) | 5 |
| ITRQ Reflection and Comfort | 5.72 (2.03; 2.00-10.00) | 22 |  | 6.16 (1.86; 2.00-10.00) | 17 |  | 4.20 (2.02; 2.00-7.00) | 5 |
| ITRQ Total Scale | 6.53 (1.88; 2.56-10.00) | 22 |  | 6.90 (1.75; 3.44-10.00) | 17 |  | 5.27 (1.92; 2.56-7.22) | 5 |
| Intervention rating | 6.95 (1.88; 3.00-10.00) | 21 |  | 7.29 (1.72; 4.00-10.00) | 17 |  | 5.50 (2.08; 3.00-8.00) | 4 |
| User-friendliness | 6.23 (2.11; 2.00-9.00) | 22 |  | 6.53 (1.66; 2.00-8.00) | 17 |  | 5.20 (3.27; 2.00-9.00) | 5 |

**Note:** iCBT = internet-based cognitive-behavioral therapy; *M* = mean; *SD* = standard deviation; *n* = number; ITRQ = Internet-Specific Therapeutic Relationship Questionnaire.

**Table S7**

**Patient satisfaction for the iCBT group, and for completers and non-completers separately in the fibromyalgia study**

|  | iCBT | |  | Completers | |  | Non-completers | |
| --- | --- | --- | --- | --- | --- | --- | --- | --- |
|  | *M* (*SD*; range) | *n* |  | *M* (*SD*; range) | *n* |  | *M* (*SD*; range) | *n* |
| ***Baseline*** |  |  |  |  |  |  |  |  |
| ITRQ Time and Attention | 7.17 (1.31; 3.50-9.25) | 33 |  | 7.28 (1.22; 3.50-9.25) | 25 |  | 6.81 (1.60; 4.00-8.50) | 8 |
| ITRQ Reflection and Comfort | 6.89 (1.29; 4.50-9.25) | 33 |  | 7.01 (1.23; 4.50-9.25) | 25 |  | 6.53 (1.50; 4.75-8.75) | 8 |
| ITRQ Total Scale | 7.03 (1.13; 4.11-8.67) | 33 |  | 7.15 (1.05; 4.11-8.67) | 25 |  | 6.68 (1.37; 4.44-8.56) | 8 |
| ***Post-intervention*** |  |  |  |  |  |  |  |  |
| ITRQ Time and Attention | 7.65 (1.57; 4.50-10.00) | 26 |  | 7.83 (1.54; 4.50-10.00) | 22 |  | 6.69 (1.59; 4.50-8.25) | 4 |
| ITRQ Reflection and Comfort | 6.49 (2.03; 2.75-9.75) | 27 |  | 6.76 (1.97; 2.75-9.75) | 23 |  | 4.94 (1.84; 3.00-6.75) | 4 |
| ITRQ Total Scale | 7.15 (1.66; 4.11-9.67) | 26 |  | 7.36 (1.65; 4.33-9.67) | 22 |  | 5.97 (1.33; 4.11-7.22) | 4 |
| Intervention rating | 6.92 (1.98; 1.00-10.00) | 26 |  | 7.32 (1.62; 4.00-10.00) | 22 |  | 4.75 (2.63; 1.00-7.00) | 4 |
| User-friendliness | 7.08 (1.70; 1.00-9.00) | 26 |  | 7.55 (0.96; 5.00-9.00) | 22 |  | 4.50 (2.65; 1.00-7.00) | 4 |

**Note:** iCBT = internet-based cognitive-behavioral therapy; *M* = mean; *SD* = standard deviation; *n* = number; ITRQ = Internet-Specific Therapeutic Relationship Questionnaire.

**Supplementary Appendix G. Sensitivity analyses in the hand osteoarthritis study and fibromyalgia study**

**Sensitivity analyses: completers-only**

*Hand osteoarthritis*

Results of the completer analysis are shown in Table S8 in Supplementary Appendix G. When including the covariates age and sex in the analyses, the results of the completer analysis for the primary outcome (pain coping) showed a stronger effect (B = 1.28; *p* = .024) at 6-week follow-up compared to the primary analysis. Similar to the primary analysis, no significant interactions at post-intervention or 3-month follow-up were found for pain coping. For the secondary outcomes, in contrast to the primary analysis, a significant interaction was found for Health Change (RAND-36) at post-intervention, showing that intervention completers reported a larger improvement in perceived health compared to a year ago than patients in the control group. Furthermore, several effects were confirmed in the completer analysis (e.g., Identity [IPQ-R] at post-intervention and Acceptance [ICQ] at 3-month follow-up) and other effects were no longer significant in the completer analysis when compared to the primary analysis (e.g., Identity [IPQ-R] at 6-week follow-up, Passive Pain Coping [PCI], and Affective Distress [MPI-DLV] at 3-month follow-up). The results of the completer analysis without the age and sex covariates were similar to the results of the completer analysis including the covariates.

*Fibromyalgia*

Results of the completer analysis are shown in Table S9 in Supplementary Appendix G. When including the covariates age and sex in the analyses, the results of the completer analysis for the primary outcome (pain coping) showed a slightly weaker effect (B = 1.26; *p* = .011) at post-intervention compared to the primary analysis. Like the primary analysis, no significant interactions at 6-week or 3-month follow-up were found for pain coping. For the secondary outcomes, in contrast to the primary analysis, the interactions of Active Pain Coping (PCI) at post-intervention and 3-month follow-up were significant in the completer analysis, showing that intervention completers reported more use of active pain coping skills than patients in the control group. Also, Emotional Representations (IPQ-R), Life Control (MPI-DLV), and Energy/Fatigue (RAND-36) at 3-month follow-up were significant in the completer analysis as opposed to the primary analysis, showing that intervention completers reported having fewer negative emotions regarding the illness, higher life control, and more energy than patients in the control group. Several effects were confirmed in the completer analysis when compared to the primary analysis (e.g., intervention completers reported better well-being [VAS] and less worrying [PCI] than controls at post-intervention and 3 month-follow-up and more retreating (PCI) than controls at 3-month follow-up). Some effects were no longer significant in the completer analysis when compared to the primary analyses (e.g., Well-being [VAS] and Treatment Control [IPQ-R] at 6-week follow-up). The results of the completer analysis without the age and sex covariates were mostly similar to the results of the completer analysis including the covariates. The main differences included Emotional Representations (IPQ-R) and Life Control (MPI-DLV) not being significant at 3-month follow-up in the completer analysis without covariates contrary to the completer analysis with covariates.

**Table S8**

**Results^a^ of linear mixed model analyses for primary and secondary outcomes of the sensitivity analysis: completers only, including covariates in the hand osteoarthritis study**

|  | Intercept | | |  | Short-term*group^b^  (T2 – T1) | | |  | Mid-term*group^b^  (T3 – T1) | | |  | Long-term*group^b^  (T4 – T1) | | |
| --- | --- | --- | --- | --- | --- | --- | --- | --- | --- | --- | --- | --- | --- | --- | --- |
|  | Est | *SE* | *p* |  | Est | *SE* | *p* |  | Est | *SE* | *p* |  | Est | *SE* | *p* |
| **Primary outcome** |  |  |  |  |  |  |  |  |  |  |  |  |  |  |  |
| Pain coping (VAS) | 6.48 | 0.49 | <.001 |  | 0.77 | 0.53 | .145 |  | 1.28 | 0.56 | **.024** |  | 0.44 | 0.58 | .441 |
| **Secondary outcomes** |  |  |  |  |  |  |  |  |  |  |  |  |  |  |  |
| **Psychological functioning** |  |  |  |  |  |  |  |  |  |  |  |  |  |  |  |
| ***Well-being*** |  |  |  |  |  |  |  |  |  |  |  |  |  |  |  |
| Well-being (VAS) | 6.66 | 0.44 | <.001 |  | 0.76 | 0.53 | .150 |  | 0.77 | 0.56 | .171 |  | -0.34 | 0.58 | .557 |
| RAND-36 |  |  |  |  |  |  |  |  |  |  |  |  |  |  |  |
| Mental Composite Score | 71.92 | 4.86 | <.001 |  | 6.46 | 4.23 | .128 |  | 1.41 | 4.48 | .753 |  | 3.40 | 4.59 | .460 |
| Emotional Well-being | 77.52 | 3.89 | <.001 |  | 5.80 | 3.27 | .078 |  | 0.24 | 3.47 | .946 |  | 6.72 | 3.55 | .060 |
| MPI-DLV |  |  |  |  |  |  |  |  |  |  |  |  |  |  |  |
| Affective Distress | 1.48 | 0.29 | <.001 |  | -0.23 | 0.29 | .424 |  | 0.53 | 0.31 | .091 |  | -0.59 | 0.32 | .065 |
| ***Coping and cognitions*** |  |  |  |  |  |  |  |  |  |  |  |  |  |  |  |
| ICQ |  |  |  |  |  |  |  |  |  |  |  |  |  |  |  |
| Helplessness | 11.54 | 0.95 | <.001 |  | 0.18 | 0.63 | .772 |  | -0.69 | 0.66 | .298 |  | 0.77 | 0.68 | .254 |
| Acceptance | 15.20 | 0.92 | <.001 |  | 1.38 | 0.80 | .088 |  | 1.09 | 0.85 | .202 |  | 2.00 | 0.87 | **.022** |
| PCI |  |  |  |  |  |  |  |  |  |  |  |  |  |  |  |
| Active Pain Coping | 24.38 | 1.44 | <.001 |  | 0.12 | 1.15 | .915 |  | 2.03 | 1.22 | .098 |  | 0.88 | 1.25 | .483 |
| Transformation | 8.28 | 0.75 | <.001 |  | -0.46 | 0.56 | .415 |  | 0.41 | 0.59 | .494 |  | 0.14 | 0.61 | .813 |
| Distraction | 10.02 | 0.84 | <.001 |  | 0.42 | 0.79 | .598 |  | 1.58 | 0.84 | .060 |  | 0.87 | 0.86 | .309 |
| Reducing Demands | 6.08 | 0.43 | <.001 |  | 0.24 | 0.48 | .613 |  | 0.07 | 0.51 | .889 |  | -0.13 | 0.53 | .810 |
| Passive Pain Coping | 35.21 | 2.60 | <.001 |  | -0.53 | 1.70 | .756 |  | -0.48 | 1.79 | .790 |  | -2.28 | 1.83 | .214 |
| Retreating | 10.85 | 1.12 | <.001 |  | -0.47 | 0.70 | .501 |  | -0.88 | 0.74 | .232 |  | -1.41 | 0.75 | .062 |
| Worrying | 15.16 | 1.41 | <.001 |  | -0.50 | 1.15 | .666 |  | -0.26 | 1.22 | .830 |  | -0.85 | 1.25 | .498 |
| Resting | 9.20 | 0.71 | <.001 |  | 0.39 | 0.53 | .462 |  | 0.61 | 0.56 | .281 |  | -0.08 | 0.58 | .895 |
| IPQ-R |  |  |  |  |  |  |  |  |  |  |  |  |  |  |  |
| Identity | 5.08 | 0.42 | <.001 |  | -0.83 | 0.38 | **.030** |  | -0.66 | 0.40 | .103 |  | -0.59 | 0.41 | .152 |
| Timeline Acute/Chronic^c^ | 1.11 | 0.09 | <.001 |  | 0.18 | 0.09 | .052 |  | -0.09 | 0.09 | .345 |  | 0.10 | 0.10 | .312 |
| Timeline Cyclical | 12.21 | 0.73 | <.001 |  | 0.17 | 0.73 | .818 |  | -0.71 | 0.78 | .365 |  | -0.22 | 0.80 | .781 |
| Consequences^d^ | 1.18 | 0.03 | <.001 |  | -0.02 | 0.02 | .450 |  | -0.04 | 0.03 | .101 |  | -0.04 | 0.03 | .183 |
| Personal Control | 17.94 | 0.89 | <.001 |  | 0.80 | 0.89 | .367 |  | 0.81 | 0.94 | .389 |  | -0.03 | 0.97 | .979 |
| Treatment Control | 14.06 | 0.68 | <.001 |  | 0.78 | 0.64 | .220 |  | 0.31 | 0.67 | .641 |  | 0.28 | 0.69 | .684 |
| Emotional Rep | 14.03 | 1.26 | <.001 |  | -1.25 | 1.02 | .220 |  | -2.01 | 1.07 | .063 |  | -3.00 | 1.10 | **.007** |
| Illness Coherence | 19.69 | 1.01 | <.001 |  | 0.22 | 0.86 | .794 |  | 0.07 | 0.91 | .941 |  | 0.79 | 0.93 | .395 |
| MPI-DLV |  |  |  |  |  |  |  |  |  |  |  |  |  |  |  |
| Life Control^c^ | 0.47 | 0.05 | <.001 |  | 0.01 | 0.05 | .818 |  | -0.05 | 0.05 | .347 |  | 0.16 | 0.05 | **.002** |
| **Physical functioning** |  |  |  |  |  |  |  |  |  |  |  |  |  |  |  |
| Pain (VAS) | 5.18 | 0.60 | <.001 |  | -0.16 | 0.52 | .766 |  | -0.43 | 0.55 | .435 |  | -0.53 | 0.57 | .346 |
| AUSCAN |  |  |  |  |  |  |  |  |  |  |  |  |  |  |  |
| Pain | 8.96 | 1.09 | <.001 |  | 0.00^e^ | 0.74 | .998 |  | 0.11 | 0.78 | .893 |  | 0.46 | 0.80 | .565 |
| Disability | 15.00 | 2.07 | <.001 |  | 0.48 | 1.20 | .687 |  | 0.78 | 1.26 | .536 |  | -1.69 | 1.29 | .192 |
| MPI-DLV |  |  |  |  |  |  |  |  |  |  |  |  |  |  |  |
| Pain Severity | 2.46 | 0.35 | <.001 |  | 0.57 | 0.31 | .066 |  | 0.50 | 0.33 | .129 |  | -0.09 | 0.34 | .782 |
| RAND-36 |  |  |  |  |  |  |  |  |  |  |  |  |  |  |  |
| Phys. Composite Score | 53.49 | 5.48 | <.001 |  | 1.41 | 3.36 | .674 |  | -0.96 | 3.54 | .788 |  | 3.68 | 3.63 | .312 |
| Physical Functioning | 69.05 | 5.83 | <.001 |  | 0.53 | 3.86 | .891 |  | -0.56 | 4.07 | .890 |  | 3.53 | 4.17 | .398 |
| Bodily Pain | 56.01 | 4.41 | <.001 |  | 6.16 | 4.04 | .129 |  | 1.28 | 4.28 | .765 |  | 1.29 | 4.39 | .770 |
| Energy/Fatigue | 57.51 | 4.53 | <.001 |  | 1.10 | 3.78 | .771 |  | 0.69 | 4.00 | .864 |  | 0.45 | 4.10 | .913 |
| Health Change | 40.76 | 5.29 | <.001 |  | 12.90 | 5.63 | **.023** |  | 7.41 | 5.98 | .217 |  | 12.88 | 6.14 | **.037** |
| **Impact on daily life** |  |  |  |  |  |  |  |  |  |  |  |  |  |  |  |
| MPI-DLV |  |  |  |  |  |  |  |  |  |  |  |  |  |  |  |
| Interference | 1.90 | 0.32 | <.001 |  | 0.09 | 0.23 | .678 |  | 0.10 | 0.24 | .667 |  | -1.00 | 0.24 | .695 |
| Support^d^ | 0.60 | 0.06 | <.001 |  | 0.04 | 0.04 | .326 |  | 0.03 | 0.04 | .493 |  | -0.06 | 0.04 | .130 |
| RAND-36 |  |  |  |  |  |  |  |  |  |  |  |  |  |  |  |
| Role Limitations due to Physical Health Problems | 29.91 | 10.42 | .006 |  | 1.12 | 8.63 | .897 |  | -4.53 | 9.14 | .621 |  | 7.70 | 9.37 | .412 |
| General Health Perceptions | 58.90 | 4.88 | <.001 |  | -1.30 | 3.35 | .698 |  | 1.58 | 3.54 | .657 |  | 3.91 | 3.63 | .282 |
| Role Limitations due to Per-sonal or Emotional Problems^c^ | 1.39 | 0.21 | <.001 |  | 0.26 | 0.24 | .283 |  | -0.24 | 0.25 | .341 |  | 0.14 | 0.26 | .597 |
| Social Functioning^c^ | 0.98 | 0.17 | <.001 |  | 0.18 | 0.18 | .327 |  | 0.23 | 0.19 | .245 |  | -0.09 | 0.20 | .659 |

**Note:** Bold face indicates a significant time-by-group interaction. EST = the estimate of the regression coefficient (i.e., for intercept: the mean of both groups at baseline, corrected for age and sex; for short-term*group, mid-term*group, and long-term*group: the change in the variable from baseline for the intervention group compared to the control group); *SE* = standard error; VAS = visual analogue scale; RAND-36 = RAND 36-item Health Survey; MPI-DLV = Multidimensional Pain Inventory-Dutch Language Version; ICQ = Illness Cognition Questionnaire; PCI = Pain Coping Inventory; IPQ-R = Illness Perception Questionnaire-Revised; AUSCAN = Australian/Canadian Hand Osteoarthritis Index.

^a^Time as three dummy variables, age, and sex were included in all models, but coefficients are not shown for these terms.

^b^The models included fixed effects of time as three dummy variables: “short-term” (post-intervention vs. baseline), “mid-term” (6-week follow-up vs. baseline), and “long-term” (3-month follow-up vs. baseline). To examine iCBT effects over time, fixed effects of interactions between group (i.e., iCBT or CAU) and timepoint were included as three dummy variables: short-term*group, mid-term*group, and long-term*group.

^c, d^Due to non-normal distributions, these variables were transformed before using them in the linear mixed model analyses in the following way: ^c^inverse and log10-transformation (negatively skewed) and ^d^log10-transformation (positively skewed). Results of the transformed variables are displayed.

^e^Estimates between -0.004 and 0.004 were rounded to 0.00.

**Table S9**

**Results^a^ of linear mixed model analyses for primary and secondary outcomes of the sensitivity analysis: completers only, including covariates in the fibromyalgia study**

|  | Intercept | | |  | Short-term*group^b^  (T2 – T1) | | |  | Mid-term*group^b^  (T3 – T1) | | |  | Long-term*group^b^  (T4 – T1) | | |
| --- | --- | --- | --- | --- | --- | --- | --- | --- | --- | --- | --- | --- | --- | --- | --- |
|  | Est | *SE* | *p* |  | Est | *SE* | *p* |  | Est | *SE* | *p* |  | Est | *SE* | *p* |
| **Primary outcome** |  |  |  |  |  |  |  |  |  |  |  |  |  |  |  |
| Pain coping (VAS) | 5.85 | 0.57 | <.001 |  | 1.26 | 0.49 | **.011** |  | 0.19 | 0.52 | .722 |  | 0.73 | 0.53 | .171 |
| **Secondary outcomes** |  |  |  |  |  |  |  |  |  |  |  |  |  |  |  |
| **Psychological functioning** |  |  |  |  |  |  |  |  |  |  |  |  |  |  |  |
| ***Well-being*** |  |  |  |  |  |  |  |  |  |  |  |  |  |  |  |
| Well-being (VAS) | 5.70 | 0.46 | <.001 |  | 1.00 | 0.44 | **.024** |  | 0.88 | 0.46 | .058 |  | 1.02 | 0.47 | **.032** |
| RAND-36 |  |  |  |  |  |  |  |  |  |  |  |  |  |  |  |
| Mental Composite Score | 49.24 | 7.14 | <.001 |  | 4.69 | 4.37 | .284 |  | 6.72 | 4.61 | .146 |  | 2.87 | 4.67 | .540 |
| Emotional Well-being | 68.08 | 6.49 | <.001 |  | 0.37 | 3.59 | .918 |  | 2.66 | 3.74 | .479 |  | -2.01 | 3.79 | .596 |
| MPI-DLV |  |  |  |  |  |  |  |  |  |  |  |  |  |  |  |
| Affective Distress | 2.66 | 0.40 | <.001 |  | -0.31 | 0.28 | .265 |  | -0.41 | 0.29 | .160 |  | -0.45 | 0.30 | .135 |
| ***Coping and cognitions*** |  |  |  |  |  |  |  |  |  |  |  |  |  |  |  |
| ICQ |  |  |  |  |  |  |  |  |  |  |  |  |  |  |  |
| Helplessness | 16.76 | 1.39 | <.001 |  | -1.08 | 0.65 | .096 |  | -0.88 | 0.67 | .189 |  | -0.60 | 0.68 | .381 |
| Acceptance | 13.54 | 1.47 | <.001 |  | 1.51 | 0.62 | **.017** |  | 0.88 | 0.65 | .177 |  | 1.89 | 0.65 | **.004** |
| PCI |  |  |  |  |  |  |  |  |  |  |  |  |  |  |  |
| Active Pain Coping | 26.51 | 1.58 | <.001 |  | 1.84 | 0.88 | **.038** |  | 1.68 | 0.91 | .068 |  | 2.00 | 0.92 | **.031** |
| Transformation^c^ | 0.92 | 0.05 | <.001 |  | 0.04 | 0.03 | .212 |  | 0.07 | 0.03 | **.027** |  | 0.04 | 0.03 | .166 |
| Distraction | 11.69 | 0.97 | <.001 |  | 0.91 | 0.52 | .080 |  | 0.98 | 0.54 | .068 |  | 1.43 | 0.54 | **.009** |
| Reducing Demands | 6.20 | 0.46 | <.001 |  | 0.70 | 0.43 | .104 |  | -0.23 | 0.45 | .617 |  | 0.26 | 0.46 | .568 |
| Passive Pain Coping | 51.46 | 3.51 | <.001 |  | -1.18 | 1.42 | .405 |  | -0.99 | 1.47 | . 501 |  | -1.78 | 1.48 | .232 |
| Retreating | 16.92 | 1.49 | <.001 |  | 0.77 | 0.63 | .223 |  | 0.96 | 0.65 | .142 |  | 1.85 | 0.66 | **.006** |
| Worrying | 20.87 | 2.11 | <.001 |  | -2.00 | 0.93 | **.033** |  | -1.77 | 0.96 | .068 |  | -3.31 | 0.98 | **<.001** |
| Resting | 13.68 | 1.06 | <.001 |  | -0.03 | 0.56 | .961 |  | -0.24 | 0.58 | .684 |  | -0.35 | 0.59 | .550 |
| IPQ-R |  |  |  |  |  |  |  |  |  |  |  |  |  |  |  |
| Identity | 7.42 | 0.83 | <.001 |  | 0.71 | 0.49 | .147 |  | 0.10 | 0.51 | .841 |  | -0.45 | 0.51 | .379 |
| Timeline Acute/Chronic^d^ | 1.11 | 0.13 | <.001 |  | -0.06 | 0.07 | .402 |  | -0.11 | 0.07 | .117 |  | -0.03 | 0.07 | .665 |
| Timeline Cyclical | 15.96 | 0.93 | <.001 |  | -0.70 | 0.62 | .258 |  | -0.48 | 0.64 | .456 |  | -0.73 | 0.65 | .264 |
| Consequences | 22.24 | 1.60 | <.001 |  | -0.45 | 0.67 | .507 |  | -1.02 | 0.69 | .142 |  | -0.74 | 0.70 | .297 |
| Personal Control | 18.74 | 1.44 | <.001 |  | 0.14 | 0.83 | .868 |  | -0.44 | 0.86 | .611 |  | -0.22 | 0.87 | .804 |
| Treatment Control | 13.98 | 1.09 | <.001 |  | -0.24 | 0.58 | .682 |  | -1.18 | 0.61 | .053 |  | -0.82 | 0.61 | .183 |
| Emotional Rep | 19.15 | 1.78 | <.001 |  | -0.99 | 0.94 | .297 |  | -1.04 | 0.98 | .288 |  | -1.97 | 0.99 | **.048** |
| Illness Coherence | 13.93 | 1.76 | <.001 |  | 0.81 | 0.78 | .300 |  | 1.24 | 0.81 | .125 |  | 1.36 | 0.82 | .098 |
| MPI-DLV |  |  |  |  |  |  |  |  |  |  |  |  |  |  |  |
| Life Control | 3.62 | 0.35 | <.001 |  | 0.21 | 0.25 | .386 |  | 0.42 | 0.25 | .099 |  | 0.52 | 0.26 | **.043** |
| **Physical functioning** |  |  |  |  |  |  |  |  |  |  |  |  |  |  |  |
| Pain (VAS) | 6.45 | 0.59 | <.001 |  | 0.12 | 0.44 | .779 |  | -0.31 | 0.46 | .501 |  | -1.29 | 0.47 | **.007** |
| MPI-DLV |  |  |  |  |  |  |  |  |  |  |  |  |  |  |  |
| Pain Severity | 3.67 | 0.40 | <.001 |  | 0.02 | 0.29 | .942 |  | 0.05 | 0.30 | .871 |  | -0.33 | 0.31 | .285 |
| RAND-36 |  |  |  |  |  |  |  |  |  |  |  |  |  |  |  |
| Physical Composite Score | 35.56 | 6.45 | <.001 |  | 5.84 | 3.16 | .066 |  | 3.22 | 3.32 | .333 |  | 9.43 | 3.37 | **.006** |
| Physical Functioning | 49.54 | 7.61 | <.001 |  | 4.83 | 3.47 | .166 |  | 2.26 | 3.65 | .537 |  | 4.95 | 3.70 | .182 |
| Bodily Pain | 38.35 | 6.27 | <.001 |  | 3.27 | 4.19 | .436 |  | -0.29 | 4.37 | .947 |  | 10.60 | 4.43 | **.018** |
| Energy/Fatigue | 32.19 | 6.04 | <.001 |  | 6.12 | 3.61 | .092 |  | 7.62 | 3.76 | **.044** |  | 8.93 | 3.81 | **.020** |
| Health Change | 35.56 | 9.64 | <.001 |  | 14.91 | 6.55 | **.024** |  | 12.91 | 6.91 | .063 |  | 14.69 | 7.01 | **.037** |
| **Impact on daily life** |  |  |  |  |  |  |  |  |  |  |  |  |  |  |  |
| MPI-DLV |  |  |  |  |  |  |  |  |  |  |  |  |  |  |  |
| Interference | 3.66 | 0.42 | <.001 |  | -0.09 | 0.19 | .637 |  | -0.24 | 0.20 | .223 |  | -0.71 | 0.20 | **<.001** |
| Support | 3.76 | 0.51 | <.001 |  | -0.13 | 0.27 | .624 |  | 0.38 | 0.28 | .178 |  | -0.03 | 0.28 | .907 |
| RAND-36 |  |  |  |  |  |  |  |  |  |  |  |  |  |  |  |
| Role Limitations due to Physical Health Problems | 18.73 | 11.20 | .099 |  | 11.30 | 7.29 | .123 |  | 4.15 | 7.69 | .590 |  | 18.70 | 7.80 | **.017** |
| General Health Perceptions^e^ | 5.95 | 0.59 | <.001 |  | 0.46 | 0.30 | .126 |  | 0.59 | 0.31 | .060 |  | 0.23 | 0.32 | .476 |
| Role Limitations due to Per-sonal or Emotional Problems | 50.31 | 13.90 | <.001 |  | 7.45 | 10.90 | .495 |  | 11.32 | 11.54 | .327 |  | 4.01 | 11.71 | .732 |
| Social Functioning | 46.10 | 8.68 | <.001 |  | 9.87 | 4.78 | **.040** |  | 9.41 | 4.98 | .060 |  | 4.58 | 5.05 | .365 |
| FIQ | 63.68 | 5.61 | <.001 |  | -3.63 | 3.08 | .240 |  | -5.25 | 3.24 | .107 |  | -6.23 | 3.29 | .060 |

**Note:** Bold face indicates a significant time-by-group interaction. EST = the estimate of the regression coefficient (i.e., for intercept: the mean of both groups at baseline, corrected for age and sex; for short-term*group, mid-term*group, and long-term*group: the change in the variable from baseline for the intervention group compared to the control group); *SE* = standard error; VAS = visual analogue scale; RAND-36 = RAND 36-item Health Survey; MPI-DLV = Multidimensional Pain Inventory-Dutch Language Version; ICQ = Illness Cognition Questionnaire; PCI = Pain Coping Inventory; IPQ-R = Illness Perception Questionnaire-Revised; FIQ = Fibromyalgia Impact Questionnaire.

^a^Time (compared to baseline) as three dummy variables, age, and sex were included in all models, but coefficients are not shown for these terms.

^b^The models included fixed effects of time as three dummy variables: “short-term” (post-intervention vs. baseline), “mid-term” (6-week follow-up vs. baseline), and “long-term” (3-month follow-up vs. baseline). To examine iCBT effects over time, fixed effects of interactions between group (i.e., iCBT or CAU) and timepoint were included as three dummy variables: short-term*group, mid-term*group, and long-term*group.

^c, d, e^Due to non-normal distributions, these variables were transformed before using them in the linear mixed model analyses in the following way: ^c^log10-transformation (positively skewed); ^d^inverse and log10-transformation (negatively skewed); and ^e^square-root transformation (positively skewed). Results of the transformed variables are displayed.

**Supplementary Appendix H. Intercepts of main linear mixed model analyses**

**Table S10**

Results^a,b^ of linear mixed model analyses for primary and secondary outcomes in the hand osteoarthritis study: intercepts

|  | Intercept | | |  |
| --- | --- | --- | --- | --- |
|  | Est | *SE* | *p* |  |
| **Primary outcome** |  |  |  |  |
| Pain coping (VAS) | 6.55 | 0.42 | <.001 |  |
| **Secondary outcomes** |  |  |  |  |
| **Psychological functioning** |  |  |  |  |
| ***Well-being*** |  |  |  |  |
| Well-being (VAS) | 6.68 | 0.39 | <.001 |  |
| RAND-36 |  |  |  |  |
| Mental Composite Score | 71.29 | 4.13 | <.001 |  |
| Emotional Well-being | 76.98 | 3.32 | <.001 |  |
| MPI-DLV |  |  |  |  |
| Affective Distress | 1.52 | 0.27 | <.001 |  |
| ***Coping and Cognitions*** |  |  |  |  |
| ICQ |  |  |  |  |
| Helplessness | 11.22 | 0.89 | <.001 |  |
| Acceptance | 15.36 | 0.81 | <.001 |  |
| PCI |  |  |  |  |
| Active Pain Coping | 23.15 | 1.26 | <.001 |  |
| Transformation | 7.79 | 0.64 | <.001 |  |
| Distraction | 9.23 | 0.72 | <.001 |  |
| Reducing Demands | 6.21 | 0.40 | <.001 |  |
| Passive Pain Coping | 34.20 | 2.25 | <.001 |  |
| Retreating | 10.45 | 0.92 | <.001 |  |
| Worrying | 14.66 | 1.24 | <.001 |  |
| Resting | 9.18 | 0.64 | <.001 |  |
| IPQ-R |  |  |  |  |
| Identity | 4.78 | 0.38 | <.001 |  |
| Timeline Acute/Chronic^c^ | 1.11 | 0.08 | <.001 |  |
| Timeline Cyclical | 12.53 | 0.62 | <.001 |  |
| Consequences^d^ | 1.17 | 0.03 | <.001 |  |
| Personal Control | 18.32 | 0.77 | <.001 |  |
| Treatment Control | 13.96 | 0.62 | <.001 |  |
| Emotional Rep | 13.82 | 1.08 | <.001 |  |
| Illness Coherence | 19.56 | 0.86 | <.001 |  |
| MPI-DLV |  |  |  |  |
| Life Control^c^ | 0.51 | 0.04 | <.001 |  |
| **Physical functioning** |  |  |  |  |
| Pain (VAS) | 4.94 | 0.51 | <.001 |  |
| AUSCAN |  |  |  |  |
| Pain | 8.43 | 0.92 | <.001 |  |
| Disability | 13.59 | 1.85 | <.001 |  |
| MPI-DLV |  |  |  |  |
| Pain Severity | 2.41 | 0.29 | <.001 |  |
| RAND-36 |  |  |  |  |
| Phys. Composite Score | 57.98 | 4.84 | <.001 |  |
| Physical Functioning | 72.32 | 4.84 | <.001 |  |
| Bodily Pain | 58.71 | 3.90 | <.001 |  |
| Energy/Fatigue | 57.16 | 3.84 | <.001 |  |
| Health Change | 44.25 | 4.68 | <.001 |  |
| **Impact on daily life** |  |  |  |  |
| MPI-DLV |  |  |  |  |
| Interference | 1.80 | 0.30 | <.001 |  |
| Support^d^ | 0.61 | 0.05 | <.001 |  |
| RAND-36 |  |  |  |  |
| Role Limitations due to Physical Health Problems | 40.78 | 9.83 | <.001 |  |
| General Health Perceptions | 59.07 | 4.09 | <.001 |  |
| Role Limitations due to Personal or Emotional Problems^c^ | 1.38 | 0.19 | <.001 |  |
| Social Functioning^c^ | 0.95 | 0.15 | <.001 |  |

**Note:** EST = the estimate of the regression coefficient (i.e., for intercept: the mean of both groups at baseline, corrected for age and sex); *SE* = standard error; VAS = visual analogue scale; RAND-36 = RAND 36-item Health Survey; MPI-DLV = Multidimensional Pain Inventory-Dutch Language Version; ICQ = Illness Cognition Questionnaire; PCI = Pain Coping Inventory; IPQ-R = Illness Perception Questionnaire-Revised; AUSCAN = Australian/Canadian Hand Osteoarthritis Index.

^a^Time (compared to baseline) as three dummy variables, age, and sex were included in all models, but coefficients are not reported.

^b^The models included fixed effects of time as three dummy variables: “short-term” (post-intervention vs. baseline), “mid-term” (6-week follow-up vs. baseline), and “long-term” (3-month follow-up vs. baseline). To examine iCBT effects over time, fixed effects of interactions between group (i.e., iCBT or CAU) and timepoint were included as three dummy variables: short-term*group, mid-term*group, and long-term*group (reported in Table 2).

^c, d^Due to non-normal distributions, these variables were transformed before using them in the linear mixed model analyses in the following way: ^c^inverse and log10-transformation (negatively skewed) and ^d^log10-transformation (positively skewed). Results of the transformed variables are displayed.

**Table S11**

Results^a,b^ of linear mixed model analyses for primary and secondary outcomes in the fibromyalgia study: intercepts

|  | Intercept | | |  |
| --- | --- | --- | --- | --- |
|  | Est | *SE* | *p* |  |
| **Primary outcome** |  |  |  |  |
| Pain coping (VAS) | 5.83 | 0.54 | <.001 |  |
| **Secondary outcomes** |  |  |  |  |
| **Psychological functioning** |  |  |  |  |
| ***Well-being*** |  |  |  |  |
| Well-being (VAS) | 5.76 | 0.44 | <.001 |  |
| RAND-36 |  |  |  |  |
| Mental Composite Score | 49.33 | 7.03 | <.001 |  |
| Emotional Well-being | 68.52 | 6.32 | <.001 |  |
| MPI-DLV |  |  |  |  |
| Affective Distress | 2.66 | 0.40 | <.001 |  |
| ***Coping and Cognitions*** |  |  |  |  |
| ICQ |  |  |  |  |
| Helplessness | 16.84 | 1.36 | <.001 |  |
| Acceptance | 13.75 | 1.43 | <.001 |  |
| PCI |  |  |  |  |
| Active Pain Coping | 26.80 | 1.58 | <.001 |  |
| Transformation^c^ | 0.93 | 0.05 | <.001 |  |
| Distraction | 11.70 | 0.96 | <.001 |  |
| Reducing Demands | 6.28 | 0.46 | <.001 |  |
| Passive Pain Coping | 51.41 | 3.39 | <.001 |  |
| Retreating | 17.04 | 1.48 | <.001 |  |
| Worrying | 20.62 | 2.02 | <.001 |  |
| Resting | 13.77 | 1.08 | <.001 |  |
| IPQ-R |  |  |  |  |
| Identity | 7.46 | 0.80 | <.001 |  |
| Timeline Acute/Chronic^d^ | 1.11 | 0.13 | <.001 |  |
| Timeline Cyclical | 15.77 | 0.93 | <.001 |  |
| Consequences | 22.26 | 1.58 | <.001 |  |
| Personal Control | 18.69 | 1.40 | <.001 |  |
| Treatment Control | 13.93 | 1.05 | <.001 |  |
| Emotional Rep | 19.09 | 1.74 | <.001 |  |
| Illness Coherence | 13.87 | 1.75 | <.001 |  |
| MPI-DLV |  |  |  |  |
| Life Control | 3.64 | 0.34 | <.001 |  |
| **Physical functioning** |  |  |  |  |
| Pain (VAS) | 6.52 | 0.57 | <.001 |  |
| MPI-DLV |  |  |  |  |
| Pain Severity | 3.69 | 0.38 | <.001 |  |
| RAND-36 |  |  |  |  |
| Physical Composite Score | 35.16 | 6.18 | <.001 |  |
| Physical Functioning | 49.64 | 7.60 | <.001 |  |
| Bodily Pain | 36.94 | 6.07 | <.001 |  |
| Energy/Fatigue | 32.12 | 5.91 | <.001 |  |
| Health Change | 37.33 | 10.18 | <.001 |  |
| **Impact on daily life** |  |  |  |  |
| MPI-DLV |  |  |  |  |
| Interference | 3.66 | 0.40 | <.001 |  |
| Support | 3.81 | 0.55 | <.001 |  |
| RAND-36 |  |  |  |  |
| Role Limitations due to Physical Health Problems | 18.33 | 10.97 | .099 |  |
| General Health Perceptions^e^ | 5.96 | 0.56 | <.001 |  |
| Role Limitations due to Personal or Emotional Problems | 51.38 | 13.76 | <.001 |  |
| Social Functioning | 44.97 | 8.38 | <.001 |  |
| FIQ | 64.36 | 5.51 | <.001 |  |

**Note:** EST = the estimate of the regression coefficient (i.e., for intercept: the mean of both groups at baseline, corrected for age and sex); *SE* = standard error; VAS = visual analogue scale; RAND-36 = RAND 36-item Health Survey; MPI-DLV = Multidimensional Pain Inventory-Dutch Language Version; ICQ = Illness Cognition Questionnaire; PCI = Pain Coping Inventory; IPQ-R = Illness Perception Questionnaire-Revised; FIQ = Fibromyalgia Impact Questionnaire.

^a^Time (compared to baseline) as three dummy variables, age, and sex were included in all models, but coefficients are not reported.

^b^The models included fixed effects of time as three dummy variables: “short-term” (post-intervention vs. baseline), “mid-term” (6-week follow-up vs. baseline), and “long-term” (3-month follow-up vs. baseline). To examine iCBT effects over time, fixed effects of interactions between group (i.e., iCBT or CAU) and timepoint were included as three dummy variables: short-term*group, mid-term*group, and long-term*group (reported in Table 3).

^c, d, e^Due to non-normal distributions, these variables were transformed before using them in the linear mixed model analyses in the following way: ^c^log10-transformation (positively skewed); ^d^inverse and log10-transformation (negatively skewed); and ^e^square-root transformation (positively skewed). Results of the transformed variables are displayed.

^f^Estimates between -0.004 and 0.004 were rounded to 0.00.

**References**

1. Andersen, J.R., Breivik, K., Engelund, I.E., Iversen, M.M., Kirkeleit, J., Norekvål, T.M., Oterhals, K., Storesund, A., 2022. Correlated physical and mental health composite scores for the RAND-36 and RAND-12 health surveys: can we keep them simple? Health Qual. Life Outcomes 20, 89.

<https://doi.org/10.1186/s12955-022-01992-0>

1. Bellamy, N., Campbell, J., Haraoui, B., Gerecz-Simon, E., Buchbinder, R., Hobby, K., MacDermid, J.C., 2002. Clinimetric properties of the AUSCAN Osteoarthritis Hand Index: an evaluation of reliability, validity and responsiveness. Osteoarthr. Cartil. 10, 863–869. <https://doi.org/10.1053/joca.2002.0838>
2. Burckhardt, C.S., Clark, S.R., Bennett, R.M., 1991. The fibromyalgia impact questionnaire: development and validation. J. Rheumatol. 18, 728–733.
3. Doyle, D.V., Dieppe, P.A., Scott, J., Huskisson, E.C., 1981. An articular index for the assessment of osteoarthritis. Ann. Rheum. Dis. 40, 75–78. <https://doi.org/10.1136/ard.40.1.75>
4. Evers, A.W.M., Kraaimaat, F.W., van Lankveld, W., Jongen, P.J.H., Jacobs, J.W.G., Bijlsma, J.W.J., 2001. Beyond unfavorable thinking: the Illness Cognition Questionnaire for chronic diseases. J. Consult. Clin. Psychol. 69, 1026–1036. <https://doi.org/10.1037/0022-006X.69.6.1026>
5. Ferwerda, M., van Beugen, S., van Riel, P.C.L.M., van de Kerkhof, P.C.M., de Jong, E.M.G.J., Smit, J.V., Zeeuwen-Franssen, M.E.J., Kroft, E.B.M., Visser, H., Vonkeman, H.E., Creemers, M.C.W., van Middendorp, H., Evers, A.W.M., 2016. Measuring the therapeutic relationship in internet-based interventions. Psychother. Psychosom. 85, 47–49. <https://doi.org/10.1159/000435958>
6. Hays, R.D., Sherbourne, C.D., Mazel, R.M., 1993. The RAND 36-Item health survey 1.0. Health Econ. 2, 217–227. <https://doi.org/10.1002/hec.4730020305>
7. Hovens, I.B., van Wilgen, C.P., van Ittersum, M.W., 2009. Normscores van de Fibromyalgia Impact Questionnaire in een Nederlandse populatie fibromyalgiepatiënten. Ned. Tijdschr. Fysiother. 119, 3–9.
8. International Council for Harmonisation of Technical Requirements for Pharmaceuticals for Human Use, 2025. Guideline for good clinical practice E6(R3). Available from: <https://database.ich.org/sites/default/files/ICH_E6%28R3%29_Step4_FinalGuideline_2025_0106.pdf>
9. Kraaimaat, F.W., Bakker, A.B.H., Evers, A.W.M., 1997. Pijncoping-strategieën bij chronische pijnpatienten: de ontwikkeling van de Pijn-Coping-Inventarisatielijst (PCI). Gedragstherapie 30, 185–201.
10. Lousberg, R., van Breukelen, G.J.P., Groenman, N.H., Schmidt, A.J.M., Arntz, A., Winter, F.A.M., 1999. Psychometric properties of the Multidimensional Pain Inventory, Dutch language version (MPI-DLV). Behav. Res. Ther. 37, 167–182. <https://doi.org/10.1016/S0005-7967(98)00137-5>
11. Moorer, P., Suurmeijer, T.P.B.M., Foets, M., Molenaar, I.W., 2001. Psychometric properties of the RAND-36 among three chronic diseases (multiple sclerosis, rheumatic diseases and COPD) in the Netherlands. Qual. Life Res. 10, 637–645. <https://doi.org/10.1023/a:1013131617125>
12. Moss-Morris, R., Weinman, J., Petrie, K., Horne, R., Cameron, L., Buick, D., 2002. The revised Illness Perception Questionnaire (IPQ-R). Psychol. Health 17, 1–16.

<https://doi.org/10.1080/08870440290001494>

1. Perneger, T.V., 1998. What's wrong with Bonferroni adjustments. BMJ, 316, 1236–1238.

<https://doi.org/10.1136/bmj.316.7139.1236>

1. Raj-Koziak, D., Gos, E., Swierniak, W., Rajchel, J.J., Karpiesz, L., Niedzialek, I., Wlodarczyk, E., Skarzynski, H., Skarzynski, P.H., 2018. Visual analogue scales as a tool for initial assessment of tinnitus severity: psychometric evaluation in a clinical population. Audiol. Neurotol. 23, 229–237. <https://doi.org/10.1159/000494021>
2. Rozental, A., Andersson, G., Boettcher, J., Ebert, D.D., Cuijpers, P., Knaevelsrud, C., Ljótsson, B., Kaldo, V., Titov, N., Carlbring, P., 2014. Consensus statement on defining and measuring negative effects of Internet interventions. Internet Interv. 1, 12–19. <https://doi.org/10.1016/j.invent.2014.02.001>
3. Sanderman, R., Arrindell, W.A., Ranchor, A.V., Eysenck, H.J., Eysenck, S.B.G., 2012. Het meten van persoonlijkheidskenmerken met de Eysenck Personality Questionnaire (EPQ). Een handleiding. Noordelijk Centrum voor Gezondheidsvraagstukken, Groningen.
4. Sendlbeck, M., Araujo, E.G., Schett, G., Englbrecht, M., 2015. Psychometric properties of three single-item pain scales in patients with rheumatoid arthritis seen during routine clinical care: a comparative perspective on construct validity, reproducibility and internal responsiveness. RMD Open 1, e000140. <https://doi.org/10.1136/rmdopen-2015-000140>
5. van der Vaart R., Worm-Smeitink M., Bos Y., Wensing M., Evers A., Knoop H., 2019. Implementing guided ICBT for chronic pain and fatigue: a qualitative evaluation among therapists and managers. Internet Interv 18, 100290. <https://doi.org/10.1016/j.invent.2019.100290>
